# Epitranscriptomics Regulation of CD70, CD80, and TIGIT in Cancer Immunity

**DOI:** 10.3390/ijms26125772

**Published:** 2025-06-16

**Authors:** Christos Panagiotis Rigopoulos, Marios Gkoris, Ilias Georgakopoulos-Soares, Ioannis Boulalas, Apostolos Zaravinos

**Affiliations:** 1 Department of Life Sciences, School of Sciences, European University Cyprus, 1516 Nicosia, Cyprus; cr211316@students.euc.ac.cy (C.P.R.); mg211332@students.euc.ac.cy (M.G.); 2 Cancer Genetics, Genomics and Systems Biology Laboratory, Basic and Translational Cancer Research Center (BTCRC), 1516 Nicosia, Cyprus; 3 Institute for Personalized Medicine, Department of Biochemistry and Molecular Biology, College of Medicine, The Pennsylvania State University, Hershey, PA 17033, USA; izg5139@psu.edu; 4 Department of Urology, General Hospital of Nikaia-Piraeus “Ag. Panteleimon”, 18454 Nikaia Piraeus, Greece; iboulalas@yahoo.gr

**Keywords:** multi-omics analysis, pan-cancer, CD70, CD80, TIGIT, RNA modifications, m^1^A, m^5^C, m^6^A

## Abstract

Tumor development is mainly marked by the gradual transformation of cells that acquire capacities such as sustained growth signaling, evasion of growth suppression, resistance to cell death, and induction of angiogenesis, achieving replicative immortality and activating invasion and metastasis. How different epigenetic alterations like m^1^A, m^5^C, and m^6^A contribute to tumor development is a field that still needs to be investigated. The immune modulators, CD70, CD80, and TIGIT, mainly regulate T-cell activation and consequently the immune evasion of tumors. Here, we explored the presence and the potential consequences of RNA modifications in these regulators in pan-cancer. Our findings highlight the critical role of the m^6^A, m^5^C, and m^1^A in regulating CD70, CD80, and TIGIT across multiple solid tumors. By combining epitranscriptomics data with functional enrichment and survival modeling, we show that RNA modification enzymes not only modulate immune-related gene expression but also serve as potential biomarkers for patient prognosis. By constructing a robust four-gene prognostic signature involving YTHDF3, RBM15B, IGF2BP2, and TRMT61A, we demonstrate that RNA modification profiles can accurately stratify patients into risk groups with distinct overall survival outcomes. The performance of this model across eight cancer types underscores the translational promise of epitranscriptomic markers in both mechanistic understanding and personalized oncology. Altogether, our study bridges the gap between the mechanistic regulation of immune checkpoints and their clinical utility, offering novel insights into how the epitranscriptome can be leveraged to improve cancer prognosis and potentially enhance immunotherapeutic strategies.

## 1. Introduction

Epigenetics comprises many biological processes around chromatin-mediated DNA template regulation [1]. The protein families that regulate epigenetics mechanisms, like DNA methylation and histone modification, are classified as erasers, writers, and readers [2]. Therefore, these complexes influence gene expression and chromatin structure. Writers are enzymes that add particular post-translational modifications (PTMs) to histones. For instance, histone methyltransferases (HMTs) attach methyl groups to histone tails, altering chromatin’s compactness. Readers are methyl-CpG-binding domain proteins (MBPs) that detect and bind to histone modifications. Afterward, they recruit additional transcriptional factors to further regulate gene expression. Finally, erasers are enzymes tasked with removing histone modifications. For example, histone demethylases eliminate methyl groups, counteracting the effects of writers and readers, enabling flexible control of chromatin [3].

DNA methylation is marked by the addition of a methyl group to the C5 of cytosine residues via a DNA methyltransferase (DNMT). As a result, normal cells can easily differentiate from cancerous or other aberrant cells [4]. Humans are provided with three conserved DNMTs, DNMT1, DNMT3A, and DNMT3B. DNMT1 is primarily responsible for maintaining the pre-existing methylation pattern during replication [5,6], while DNMT3 subtypes take part in de novo methylation and non-cytosine/guanine (CpG) methylation regulating the biological processes during embryonic development, cell differentiation, and cancer cell survival [7]. Notably, the potential reversibility of the activity of DNMTs could serve as an appealing target for therapeutic interventions.

Another field of epigenomics is epi-transcriptomics, which refers to RNA modifications. RNA modifications are distinct chemical marks to the bases or the ribose sugar in the RNA molecules. The first discovered molecule was pseudouridine (Ψ) [8]. The rapid increase in the RNA modification field is due to the comprehension that RNA can be directly functional on gene expression, via non-coding RNAs (ncRNAs), like long-non-coding RNA (lncRNA), and microRNA (miRNA) [9,10].

The most common RNA modification is the methylation of adenosine at position 6, commonly known as N6-methyladenosine (m^6^A). It is predominantly found in mRNA, long-intergenic ncRNAs (lincRNAs), and rRNA [11,12,13,14]. This modification is catalyzed by the methyltransferase-like 3 (METTL3)–METTL14 complex. METTL3 serves as the main catalytic component, and METTL14 acts as the RNA-binding subunit that recognizes the component [15]. This type of methylation seems to be the most abundant mRNA methylation, and, as a result, it influences mRNA steadiness, splicing, and translation [11,16]. The METTL3–METTL14 complex preferentially methylates internal sites in mRNAs, particularly near stop codons, within 3′ untranslated regions (3′UTRs), and in long exons.

The existence of m^6^A is also crucial for the function of ncRNAs, like the X-inactive specific transcript (XIST), which plays an important role in the maintenance of random X inactivation of mammalian cells [17,18]. Notably, it has been found that this methylation can be reversed via two distinct molecules: the fat mass and obesity-associated protein (FTO) and the AlkB homolog 5 (ALKBH5) [19,20]. The m^6^A modification is recognized by a great number of readers, like members of the YTH domain-containing family, heterogeneous nuclear ribonucleoproteins A2/B1 (HNRNPA2B1), and many more enzymes [19,21]. This fact empowers the already established position of m^6^A as a regulator of many molecular pathways that lead or connect with carcinogenesis [22].

RNA 5-methylcytosine (m^5^C) is another RNA modification present in rRNA, tRNA, mRNA, and ncRNA. In tRNA, it seems to play a crucial part in the integrity of the RNA structure and aids in the accuracy of the translation. In the context of rRNA, the loss of m^5^C at position 2278 has been linked to the translational readthrough of stop codons [23,24,25]. m^5^C appears to play an essential role in the nuclear export of mature mRNAs, catalyzed by an m^5^C reader, called ALYREF [26]. The enzymes that contribute to this type of RNA modification mainly consist of members of the NSUN family, NSUN1 to NSUN7.

Another RNA modification is the methylation of adenosine at position 1 (m^1^A). It has mainly been studied in tRNAs and is preferentially located in the 5′UTR [27]. The main methyltransferase that operates during the m^1^A is the TRMT family, consisting of TRMT6, TRMT61A, TRMT10C, and TRMT61B. The YTH family has also shown signs of binding to m1A with lower affinity, particularly YTHDF2 [28].

CD70 (Cluster of Differentiation 70) is a member of the Tumor Necrosis Factor (TNF) family. It can be found mainly in activated dendritic cells (DC), B cells, T cells, and NK cells. It can also function as a transmembrane receptor when binding with the CD27 molecule to act as a costimulatory signal. This co-stimulation is essential for the proper activation of T cells [9,10]. Given CD70’s role in tumor growth and immune evasion, understanding how RNA modifications affect its expression and function could provide insights into novel therapeutic methods. For instance, N6-methyladenosine (m^6^A) modifications might influence CD70 mRNA stability or translation efficiency, thus modulating its protein levels on the cell surface. Targeting those enzymes, which add or remove these modifications, might be beneficial on controlling CD70 expression, enhancing anti-tumor immune responses [29,30].

CD80, also known as B7-1, is a molecule that belongs to the immunoglobulin superfamily (IgSF) and also exhibits costimulatory activity [31]. It is predominantly expressed as a dimer on the surface of DC, macrophages, and B and T cells [32], and it can bind to its receptor CD28 on T cells, enhancing cytokine production [33,34]. However, CD80 can also bind to CTLA-4 with higher affinity. CTLA-4 exhibits inhibitive capacity and can ease tumor evasion [35,36]. CD80’s role in the regulation of tumor immunity via RNA modifications has not been clarified yet. On the other hand, a lot of data have been published regarding the influence that RNA modifications have on CTLA-4 immunotherapies. Given CD80’s competitive relationship with CTLA-4, we can virtually predict the potential influence of investigating RNA modifications in CD80. Many studies have confirmed the association between RNA modifications and CTLA-4 expression. These changes have also been found to predict the outcome of CTLA-4 blockade immunotherapy [37,38,39,40]. Consequently, it is of paramount importance to control the level of CTLA-4, via the upregulation of CD80.

RNA modifications could potentially elevate CD80 surface expression on APCs and subsequently create a shift towards the CD80-CD28 interactions, thus enhancing T-cell activation. Moreover, m^5^C can affect alternative splicing. So, if m^5^C affects the alternative spicing of CD80, it could lead to the creation of a new CD80 isoform with decreased affinity for CTLA-4, resulting in T-cell co-stimulation [41]. On the other hand, m^6^A-modified CTLA-4 has been correlated with increased expression of CTLA-4 expression in gliomas, implicating that some tumors may exploit RNA modifications to gain a competitive advantage towards immune suppression rather than T-cell activation [42]. Therefore, targeting those epitranscriptomics mechanisms that govern the CD80-CTLA-4 competition could optimize immunotherapy strategies and reinforce anti-tumor responses.

In addition, TIGIT is a member of the PVR-like proteins. It is expressed in NK cells, in both CD4+ and CD8+ T cells, and in T regulatory cells (Tregs) [43,44,45,46]. It can bind to three distinct ligands, CD155, CD112, and CD113 with CD155 being the most common [47]. Different studies have revealed the emerging role of TIGIT in cancer immunotherapy, either as monotherapy or dual immune checkpoint inhibition (ICI) therapy [48,49,50]. There are no known data about the correlation of RNA modifications and TIGIT, but many studies have confirmed that RNA methylation regulates tumor immune evasion. The molecules that promote this immune evasion are mainly the immune checkpoints, PD-1/PD-L1, CTLA-4, and TIGIT. RNA methylation is involved pivotally in regulating tumor immunity, exerting both immunosuppressive and immunostimulant capacities. Different m^6^A markers are associated with the expression of PD-1 and PD-L1. Since TIGIT has a similar function to PD-1, it is critical to investigate the influence that RNA modifications have on it [51].

The most potent way to target most cancer cells is through harnessing the immune system. Immune cells can play an essential role in repressing tumor growth, but, unfortunately, most of them found within the tumor microenvironment (TME) either malfunction or are exhausted. The intricate regulation of immune checkpoints is essential for maintaining immune homeostasis and facilitating effective anti-tumor responses. Emerging evidence shows that RNA modifications, particularly m^6^A, play a pivotal role in modulating the expression and function of these checkpoints, therefore, influencing the immunotherapy efficacy [29,30].

RNA modifications can influence the function of different types of immune cells. In T cells, via the METTL3 enzyme, T cells remain compact, resulting in their differentiation and the specialization of naïve T cells via the IL-7/STAT5/SOCS axis [52]. ALKBH5 molecules also exhibit a fundamental role in immune regulation as they inhibit the development of γδ T cells [53]. YTHDF2 reader participates in the modulation of Treg cells in the TME, resulting in their survival and functionality, via the TNF-NF-kB pathway [54]. In tumor-associated macrophages (TAM), METTL3 can induce the M1 antitumor state of macrophages by increasing the compactness of STAT1 mRNA [55]. In DC, METTLE3 has been found to accelerate the development and maturation of DC through m^6^A methylation. Moreover, RNA modifications can also influence the expression of immune checkpoints, influencing the effectiveness of ICIs. Alterations, in m^6^A levels, can affect the expression of these checkpoints on both tumor and immune cells, therefore, modulating the TME. METLLE3-associated m^6^A modification has been found to disrupt the activation of PD-L1. Consequently, there has been a decrease in PD-L1 expression levels, making tumor cells more susceptible to immune attack and improving the efficacy of ICIs [7]. Unfortunately, METTLE3-mediated m^6^A methylation can also exhibit tumor-progressive capacities, under particular circumstances in non-small-cell lung carcinoma [56]. In addition, METTL3 influences and empowers the transcriptional activation of PD-L1 mRNA in the context of breast cancer [57]. Upregulated expression of PD-L1 has also been expressed in colorectal cancer via the m6A-modified IFIT1 [58]. In these cases, the upregulation of PD-L1 expression via increased m6A modification contributes to immune evasion. In addition, Wang et al., 2025 [59], proved that RNA modification profiles have the potential to serve as biomarkers for predicting responses to ICI treatment. The researchers found that specific patterns of m^6^A modifications in tumors have been correlated with distinct immune phenotypes and clinical outcomes. Example given, high m^6^A methylation may exhibit an immune-excluded phenotype, mainly characterized by the presence of immune cells at the ICIs. These data could prove beneficial for guided personalized treatment strategies [60]. However, no relationship has been found between any type of RNA modification and TIGIT. Thus, the urge to unveil RNA modifications’ influence in TIGIT seems greater. Moreover, given the importance that immune evasion has in the context of cancer progression and survival, we questioned whether RNA modifications regulate the expression/stability of these immune checkpoints and how does this influence affect immune evasion in cancer.

Despite rapid advancements in understanding the above-mentioned RNA modifications, several barriers continue to hinder their clinical application in cancer immunotherapy. One major challenge is the limited specificity of RNA modification enzymes—many “writers”, “erasers”, and “readers” influence broad transcriptomic landscapes, which raises concerns about their off-target effects and unintended immunomodulation. This lack of specificity complicates the development of targeted therapies that avoid systemic toxicity [61]. Furthermore, context-dependence remains a critical limitation. The m^6^A-related effects are usually tumor-specific and influenced by the TME, making it difficult to generalize findings across different cancers [62]. Another major translation hurdle is drug delivery. Although promising RNA-targeting approaches (i.e., CRISPR-Cas13 and antisense oligonucleotides) have been developed, they face pharmacokinetic challenges, such as instability in circulation, immunogenicity, and poor tumor-specific uptake [63]. Finally, the dynamic interplay between RNA modifications and other epigenetic mechanisms introduces additional layers of regulatory complexity that may cofound therapeutic responses or promote resistance [64]. These issues collectively highlight the need for more selective, context-aware, and deliverable RNA-modifying agents before they can be effectively translated into clinical oncology. Moreover, despite growing evidence that RNA modifications shape immune cell function and tumor immune evasion, their regulatory influence on immune checkpoint molecules such as CD70, CD80, and TIGIT remains largely unexplored. Moreover, the extent to which these epitranscriptomic alterations contribute to patient prognosis across diverse cancer types is not yet fully understood.

Therefore, in this study, we systematically investigate the expression patterns, functional associations, and prognostic value of m^6^A, m^5^C, and m^1^A RNA modification regulators in the context of CD70, CD80, and TIGIT across solid tumors. We hypothesize that epitranscriptomic modifications modulate the expression of these three immune checkpoints in a cancer-type-specific manner and that specific RNA modification enzymes could serve as prognostic biomarkers and potential therapeutic targets in cancer immunotherapy.

## 2. Results

### 2.1. Differential Expression in Pan-Cancer

**Figure 1:**
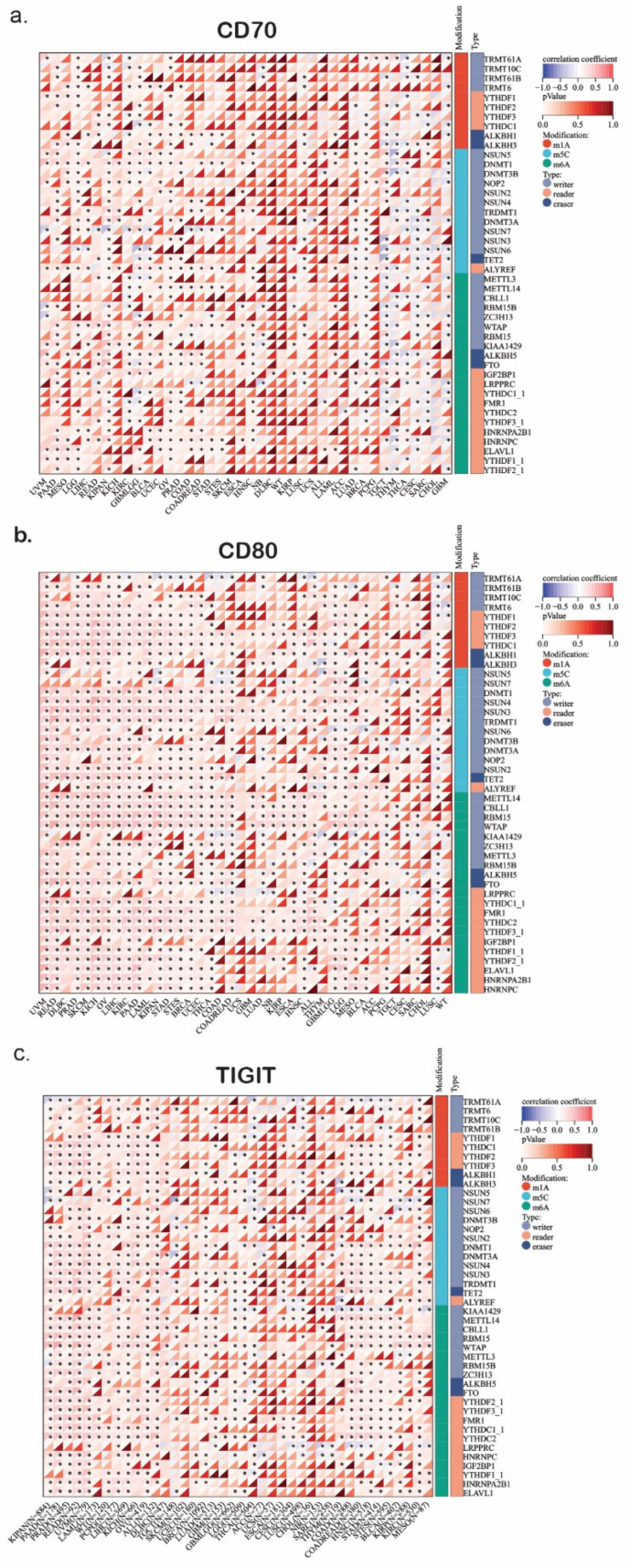
The heatmap shows the profile of correlations between RNA methylation genes (writers, readers, and erasers) and the expression of CD70 (**a**), CD80 (**b**), and TIGIT (**c**) in pan-cancer. *, *p* < 0.05.

We noticed a widespread dysregulation of RNA modification enzymes across multiple cancer types, focusing on the regulation of the immune checkpoints CD70, CD80, and TIGIT (Figure 1a–c). These findings can delve into the way that epitranscriptomics changes may modulate tumor immune evasion, immune checkpoint expression, and ultimately favor cancer progression. We then discriminated our genes based on their role in the RNA modification.

#### 2.1.1. m^6^A Writers and Their Role in CD70/CD80 Regulation

METTL3 and METTL14 are two markers that install RNA modifications by recognizing the sequence and structure of substrate RNAs, interacting with the transcriptional machinery or RNA-binding proteins (RBPs). We found them to be upregulated in LUAD, BLCA, and BRCA (Figure 1a–c). This overexpression may suggest an altered m^6^A modification landscape in these tumors, though functional m^6^A levels would require direct biochemical validation. Given that m^6^A methylation plays a crucial role in stabilizing mRNA transcripts and promoting translation efficiency, the hyperactivity of these m^6^A writers could upregulate immune-related genes, including CD70, CD80, and TIGIT. Previous studies have suggested that METTL3-mediated m6A methylation can stabilize the immune checkpoint [65,66,67].

Furthermore, WTAP upregulation in LUAD and STAD suggests that tumors with elevated m^6^A writer expression may influence the immune landscape. Since CD70 and CD80 are involved in T-cell activation and immune signaling, their expression could potentially be modulated through m^6^A-dependent mechanisms. However, whether WTAP or METTL3 directly regulate these immune checkpoints remains unclear and requires functional validation through biochemical or mechanistic assays.

#### 2.1.2. m^6^A Erasers and Their Role in Immune Regulation and Evasion

The m^6^A demethylases FTO and ALKBH5 play a key role in regulating transcript stability by removing methylation marks and influencing immune checkpoint gene expression. Our analysis revealed a strong upregulation of FTO in LUAD and PRAD, which could result in destabilization of key mRNA transcripts regarding the immune activation (Figure 1a–c). Notably, FTO has been linked to immune evasion mechanisms by downregulating the expression of immune-stimulating genes, implicating that FTO overexpression in lung and prostate cancers may impair CD70/CD80-mediated immune activation [68,69], though direct casual effects on CD70/CD80 require experimental confirmation. Reversely, ALKBH5 is significantly downregulated in ESCA and HNSC (Figure 1a–c). ALKBH5’s role is to prevent the RNA decay by removing m6A modifications. This suggests that its downregulation may result in the degradation of immune checkpoint regulations such as CD70, CD80, and TIGIT. Consequently, tumors with low-ALKBH5 expression probably have reduced immune activation, suppressing anti-tumor immune responses. Thus, the loss of ALKBH5 function in these specific cancer types may represent a novel mechanism through which tumors evade immune recognition by lowering the expression of co-stimulatory molecules like CD70/CD80, thus suppressing T-cell activation, though this mechanism remains still hypothetical without any functional evidence.

#### 2.1.3. m^6^A Readers and the Regulation of Immune Checkpoints

YTHFD1 and YTHFD2 are m^6^A-binding proteins, the role of which focuses on RNA stability and translation regulation [70]. Our findings highlight that YTHDF1 overexpression in LUAD and LUSC may be associated with enhanced translation of m6A-modified transcripts, although functional effects should be confirmed experimentally. In contrast, YTHFD2 is significantly downregulated in BRCA and PRAD, potentially leading to prolonged stability of immune-related mRNAs (Figure 1a–c). Our findings suggest that m^6^A readers might selectively regulate immune checkpoint expression, affecting CD70/CD80 dynamics in various cancer types. In addition, IGF2BP1 is an oncofetal RNA-binding protein that is strongly upregulated in PRAD and LUAD, where it is known to stabilize oncogenic transcripts, including those related to immune checkpoint signaling. IGF2BP1’s role in promoting transcript stability raises the possibility that this upregulation could extend the half-life of CD70/CD80 mRNAs, potently influencing their availability on the cell surface and altering T-cell responses, which warrants further investigation to validate these effects [71].

#### 2.1.4. TET2 Downregulation and Its Impact on TIGIT-Associated Immune Suppression

TET2 is a demethylase involved in 5-hydroxymethylation (5h^m^C), which plays a crucial role in epigenetic reprogramming and immune cell function [72]. Our findings revealed that TET2 is significantly downregulated in KIRC and COAD (Figure 1a–c). This status of TET2 may influence immunosuppressive signaling pathways, although these conclusions require further epigenetic and functional validation. Importantly, TIGIT is a well-known inhibitory immune checkpoint receptor expressed mainly on T cells and NK cells, which competes with CD226 for binding to CD155, ultimately leading to immune suppression. Consequently, this downregulation may contribute to decreased activation of anti-tumor immune responses via suppression of genes related to TIGIT signaling. Thereby, low TET2 expression might exhibit enhanced immune evasion via upregulating the TIGIT ligands, reinforcing the immune-suppressive phenotype [73,74].

### 2.2. Functional Mechanisms and Roles of m^6^A/m^5^C/m^1^A Genes

**Figure 2:**
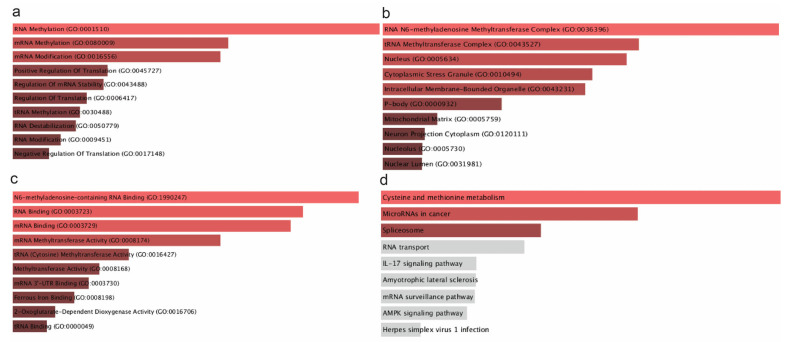
GO biological process (**a**), molecular function (**b**), cellular component (**c**), and KEGG pathway analysis (**d**) of the m^6^A/m^5^C/m^1^A genes. Τhe bar graphs are sorted by the combined score. The length of each bar represents the significance of the corresponding term. The brighter the color of the bar, the more significant the corresponding term is.

Using GO annotation, we uncovered the associated mechanisms and the potential roles of the m^6^A/m^5^C/m^1^A genes (Figure 2). As expected, the most dominant biological processes that these genes participate in are RNA methylation/mRNA methylation, mRNA stability regulation, RNA destabilization, and tRNA methylation.

RNA and mRNA methylation are present mainly via the m^6^A modifications, suggesting that m^6^A may influence immune checkpoint regulation. However, direct evidence of this functional relationship remains to be shown. This has a direct impact on CD70/CD80/TIGIT. Moreover, the regulation of RNA stability are crucial determinants of immune checkpoint expression (Figure 2a). This regulation is facilitated by the overexpression of YTHDF1 and the downregulation of YTHDF2. Additionally, IGF2BP1 is abundant in RNA stability pathways, suggesting that it may protect TIGIT mRNA from degradation and thus prolonging and reinforcing its immune-suppressive function, though this functional consequence remains speculative without validation [75]. tRNA modifications are critical for maintaining the fidelity and efficiency of protein synthesis (Figure 2a). Alterations in tRNA methylation can result in aberrant protein translation, which could probably influence the synthesis of immune checkpoint genes, like CD70, CD80, and TIGIT [76].

Regarding the cellular components in which these genes are localized, the top enriched GO terms suggest that these enzymes primarily function in nuclear and cytoplasmic compartments, influencing m^6^A RNA methylation, RNA stability, and translation control. In the number one spot is the RNA N6-methyladenosine (m^6^A) methyltransferase complex (Figure 2b). Key components of this compartment are METTL3, METTL14, WTAP, and RBM15. It is present mainly in the nucleus, where it modifies immune-related transcripts, potentially influencing checkpoint expression and immune evasion [77]. Nucleus and intracellular membrane-bounded organelles are the second site, essential for the localization of these enzymes. Nucleus is the main compartment for RNA modification, where all these enzymes, including m^6^A writers, RBPs, and TET2 operate. Ultimately, we conducted GO molecular function enrichment analysis, which highlighted the enzymatic and binding activities of RNA modification-related proteins in cancer, focused mainly on m^6^A RNA binding, methylation, and translation regulation. These molecular functions verify the critical role of these enzymes in modulating immune checkpoint expression.

### 2.3. Molecular Pathways and Protein-to-Protein Interaction Network

**Figure 3:**
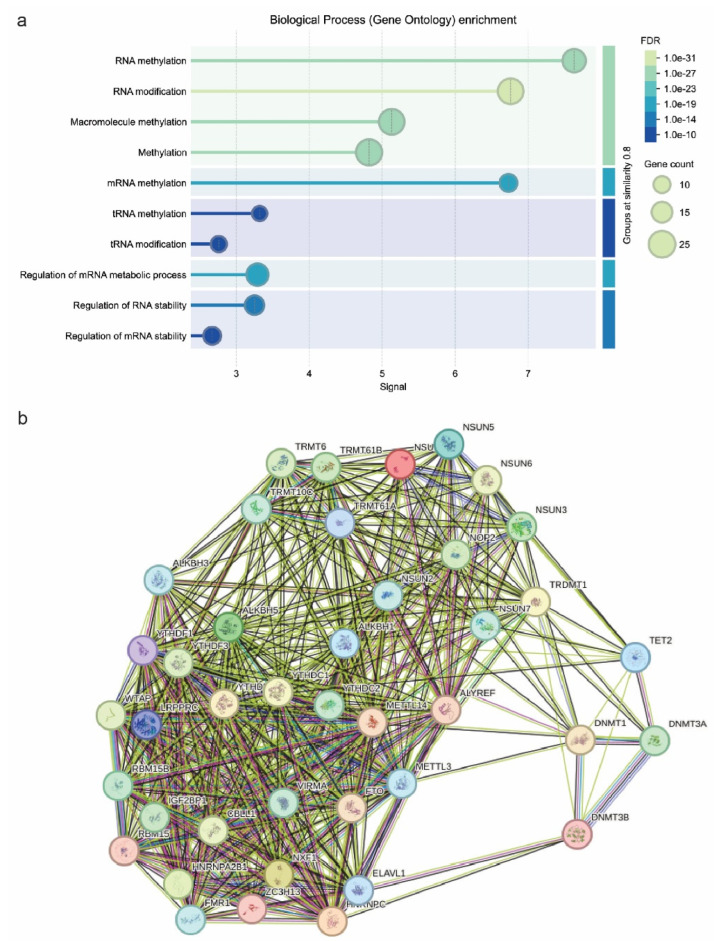
GO biological process enrichment (**a**) and pathway network analysis using STRING (**b**). In the STRING network, evidence view (grey) corresponds mainly to interactions obtained from text-mining sources. The remaining interaction colors are based on published experimental results, with Green representing activation; Red, inhibition; Blue, binding; Purple, post-translation modified; Black, reaction; and Mustard, expression. If the directionality of the effect is known, this is indicated by the symbol at the end of the edge next to the protein that is acted upon. Down-regulation is a red bar and up-regulation is a green arrow. denotes genes identified to carry de novo mutations.

We then extracted data from the KEGG database, regarding the distinct molecular pathways that these enzymes take part in. The results implicated a significant involvement of epitranscriptomic mechanisms in immune checkpoint expression and tumor immune evasion. Our KEGG analysis revealed four dominant pathways enriched in RNA modification-related genes: IL-17 signaling, microRNAs in cancer, spliceosome, and cysteine and methionine metabolism. These pathways are closely linked to the functions of CD70, CD80, and TIGIT. Specifically, IL-17 signaling has been shown to regulate the expression of CD70 and CD80 in antigen-presenting cells while promoting TIGIT-mediated suppression in the tumor microenvironment, depending on cytokine context (Figure 3b). Cysteine and methionine metabolism is a path that mainly affects RNA methylation. Methionine is the precursor of S-adenosylmethionine (SAM), the universal methyl donor in m^6^A RNA methylation. Consequently, dysregulation of this pathway may influence global RNA methylation patterns, like m^6^A levels, indirectly affecting immune checkpoint mRNA stability and translation. This pathway could be a potent immune target as it can promote immune responses against cancer [78].

MicroRNAs (miRNAs) are key post-transcriptional regulators of gene expression, including immune checkpoints. It has been found that miRNA biogenesis and function are closely related to RNA modifications such m^6^A. In the context of CD70/CD80, upregulation of miRNAs targeting these molecules could act as suppressors, impairing their immune activation. Upregulation of these miRNAs, potentially stabilized by IGF2BP1 or suppressed by YTHDF2, could repress immune checkpoint gene expression and promote immune evasion. However, such a functional relationship needs to be confirmed experimentally. Conversely, tumors may exploit miRNAs to enhance TIGIT-mediated immune suppression [79,80]. In addition, spliceosome regulates alternative splicing, impacting immune-related gene expression. Aberrant splicing patterns in cancer may lead to altered immune checkpoint isoforms. Dysregulated splicing of CD70/CD80 may alter their ability to activate T cells. In the context of TIGIT molecule, abnormal TIGIT splicing could enhance its immune-suppressive effects in tumors [81,82]. The final pathway associated with these enzymes is the IL-17 signaling pathway. IL-17 plays a dual role in cancer by promoting inflammation while also fostering an immunosuppressive environment. M6A modifications tend to regulate cytokine mRNAs, influencing IL-17 signaling. IL-17 signaling is closely associated with CD70/CD80/TIGIT molecules. In particular, increased IL-17 signaling may promote CD70/CD80 expression, modulating T-cell responses. IL-17 could also be exploited by TIGIT-expressing tumors to suppress anti-tumor immunity. Thus, IL-17-m6A-interactions could be a potent target for improving immune checkpoint blockade therapies [76,83]. Pathway network analysis revealed dense interactions between RNA modification enzymes, spotlighting their cooperative role in immune checkpoint regulation. We considered the METLL3–METTL14–WTAP complex as a central hub, suggesting that m6A deposition stabilizes immune checkpoint mRNAs, sustaining tumor immune evasion. IGF2BP1 and YTHDF1 cluster within RNA-binding networks, reinforcing their role in checkpoint translation regulation (Figure 3b). In addition, the interaction between DNMTs and TET2 supports an epigenetic mechanism governing immune checkpoint gene expression, with TET2 loss potentially silencing pro-immune genes. Finally, RNA splicing regulators (HNRNPC and HNRNPA2B1) were tightly integrated into these networks, implicating alternative splicing as a modulator of CD70/CD80/TIGIT function. These findings reveal a complex interplay between RNA modifications and immune checkpoint expression, suggesting that targeting RNA modification pathways might provide new therapeutic strategies in cancer immunotherapy [30,65,84,85].

### 2.4. Construction of a Prognostic Signature Based on RNA Modification Regulators

**Figure 4:**
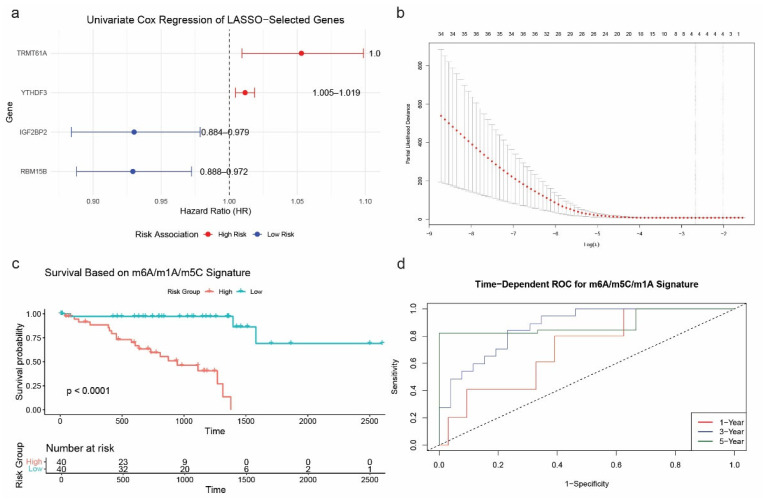
Prognostic modeling of the m6A/m5C/m1A gene signature. (**a**) Univariate Cox regression analysis of the four selected RNA modification genes across eight TCGA cancer types. (**b**) LASSO regression and cross-validation plot identifying the optimal λ value and gene subset. (**c**) Kaplan–Meier survival curves comparing overall survival between high- and low-risk groups (log-rank *p* < 0.0001). (**d**) Time-dependent ROC curves for predicting 1-, 3-, and 5-year overall survival (AUC = 0.7091, 0.8719, and 0.8879, respectively).

To identify prognostic RNA modification genes in a pan-cancer context, we performed univariate Cox regression analysis across eight TCGA cancer types (BLCA, BRCA, CESC, COAD, GBM, HNSC, KIRC, and KIRP). Several genes from the m6A/m5C/m1A regulatory families showed significant associations with overall survival (OS). Notably, YTHDF3 (HR = 1.0117, *p* = 0.0010), RBM15B (HR = 0.9292, *p* = 0.0015), IGF2BP2 (HR = 0.9302, *p* = 0.0053), and TRMT61A (HR = 1.0531, *p* = 0.0166) emerged as significant prognostic markers. To further refine these predictors, we applied LASSO (Least Absolute Shrinkage and Selection Operator) regression to construct a robust prognostic model. This method selected the same four genes for inclusion in the final risk score formula, optimized via cross-validation. The resulting model effectively stratified patients into high- and low-risk groups based on the expression of these RNA modification regulators (Figure 4a,b).

Kaplan–Meier survival analysis confirmed the model’s ability to discriminate between prognostic groups, showing significantly shorter overall survival in the high-risk group compared to the low-risk group (*p* < 0.0001; Figure 4c). In addition, time-dependent ROC curve analysis demonstrated excellent predictive performance of the model, with AUCs of 0.7091 (1-year), 0.8719 (3-year), and 0.8879 (5-year) (Figure 4d). Notably, the 5-year survival was assessed only by ROC analysis.

### 2.5. Nomogram Construction for Individualized Survival Prediction

**Figure 5:**
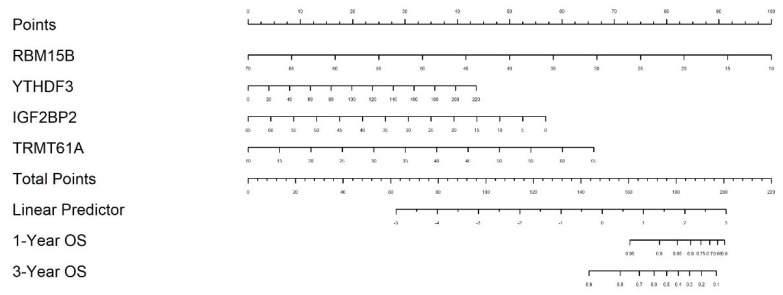
Nomogram for predicting overall survival based on the 4-gene signature. The nomogram integrates the expression of YTHDF3, RBM15B, IGF2BP2, and TRMT61A to predict 1-year and 3-year overall survival in patients across eight TCGA cancer types.

Using the four-gene signature (YTHDF3, RBM15B, IGF2BP2, and TRMT61A), we constructed a nomogram to estimate 1-year and 3-year overall survival probabilities in patients from the eight selected TCGA cancer types. The nomogram visually integrates the expression levels of each gene into a quantitative score that can be mapped to survival probabilities, offering a clinically applicable tool for personalized prognosis (Figure 5).

### 2.6. Nomogram Validation and Clinical Utility Evaluation

**Figure 6:**
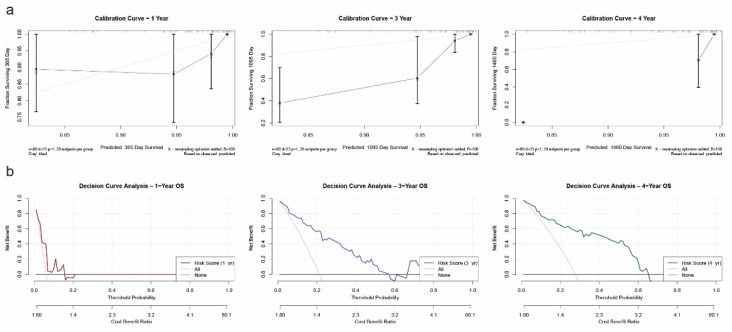
Validation of the prognostic nomogram. (**a**) Calibration curves for 1-year, 3-year, and 4-year overall survival, showing strong concordance between predicted and observed outcomes. (**b**) Decision curve analysis (DCA) for 1-year, 3-year, and 4-year OS prediction, demonstrating clinical net benefit of the model across a range of threshold probabilities.

Calibration curve analysis revealed strong agreement between predicted and observed OS outcomes at both 1-year and 3-year time points. The calibration lines closely followed the 45-degree ideal reference line, indicating high prediction accuracy (Figure 6a). Internal validation using 100 bootstrap resampling further confirmed the reliability of the nomogram model. To evaluate clinical utility, decision curve analysis (DCA) was performed. For both 1-year and 3-year predictions, the nomogram yielded a higher net benefit compared to the “treat all” and “treat none” strategies across a wide range of threshold probabilities (Figure 6b), confirming the model’s value in guiding clinical decision-making. Due to limited long-term follow-up data, 5-year predictions were not included in the calibration or decision curve analyses.

### 2.7. Cancer-Specific Prognostic Models Across Eight TCGA Cancer Types

To address tumor-specific heterogeneity and validate the generalizability of our findings, we performed separate LASSO Cox survival modeling for each of the eight TCGA cancer types, individually: BLCA, BRCA, CESC, COAD, GBM, HNSC, KIRC, and KIRP. This allowed us to construct gene-specific risk signatures tailored to each cancer type and evaluate their prognostic accuracy in a disease-specific context. For each cancer, we report the selected RNA modification-related genes, Kaplan–Meier survival analysis, ROC performance, nomogram construction, calibration accuracy, and decision curve analysis. This per-cancer stratification offers deeper biological and clinical insights compared to pan-cancer aggregation.

#### 2.7.1. Bladder Urothelial Carcinoma (BLCA)

**Figure 7:**
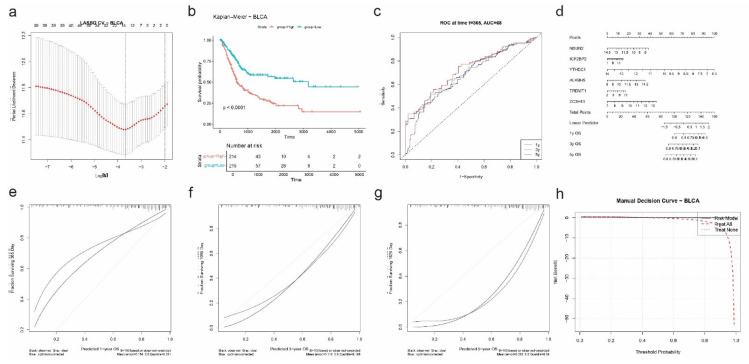
Prognostic model construction and validation for BLCA based on RNA modification-related genes. (**a**) LASSO Cox regression with 10-fold cross-validation identifies the optimal log(λ) for gene selection. (**b**) Kaplan–Meier survival curve comparing overall survival between high-risk and low-risk groups (*p* < 0.0001). (**c**) Time-dependent ROC curves at 1, 3, and 5 years show predictive performance of the risk model (AUC = 0.68 at 1 year). (**d**) Nomogram integrating expression of SIX selected genes (NSUN2, IGF2BP2, YTHDC1, ALKBH5, TRDMT1, and ZC3H13) to estimate 1-, 3-, and 5-year overall survival (OS) probabilities. (**e**–**g**) Calibration plots for 1-year (**e**), 3-year (**f**), and 5-year (**g**) OS demonstrate agreement between predicted and observed outcomes. (**h**) Decision curve analysis (DCA) evaluates net benefit of the risk model versus treat-all or treat-none strategies across threshold probabilities.

To evaluate the prognostic relevance of RNA modification regulators in bladder cancer (BLCA), we performed univariate Cox regression followed by LASSO Cox modeling. The LASSO regression, optimized using 10-fold cross-validation (Figure 7a), selected six genes: *NSUN2*, *IGF2BP2*, *YTHDC1*, *ALKBH5*, *TRDMT1*, and *ZC3H13* as the most predictive for overall survival in BLCA patients. These genes span m^6^A, m^5^C, and m^1^A pathways, highlighting diverse epitranscriptomic mechanisms.

A risk score was computed for each patient, and Kaplan–Meier analysis demonstrated a significant survival difference between high- and low-risk groups (*p* < 0.0001; Figure 7b). High-risk patients exhibited markedly shorter overall survival. Time-dependent ROC curve analysis showed good predictive accuracy with an AUC of 0.68 at 1 year (Figure 7c).

A nomogram incorporating the six genes was constructed to predict 1-, 3-, and 5-year overall survival (Figure 7d). The nomogram assigned weighted scores to each gene, which were summed to estimate survival probabilities. Calibration plots showed strong agreement between predicted and observed outcomes at all three timepoints (1y: |error| = 0.18; 3y: |error| = 0.18; 5y: |error| = 0.27) (Figure 7e).

Finally, decision curve analysis (DCA) confirmed the clinical benefit of the risk model over “treat all” and “treat none” strategies across a wide range of threshold probabilities (Figure 7f). These results support the utility of RNA modification-based signatures in stratifying prognosis among BLCA patients and underscore the translational potential of epitranscriptomic biomarkers in this cancer type.

#### 2.7.2. Breast Invasive Carcinoma (BRCA)

**Figure 8:**
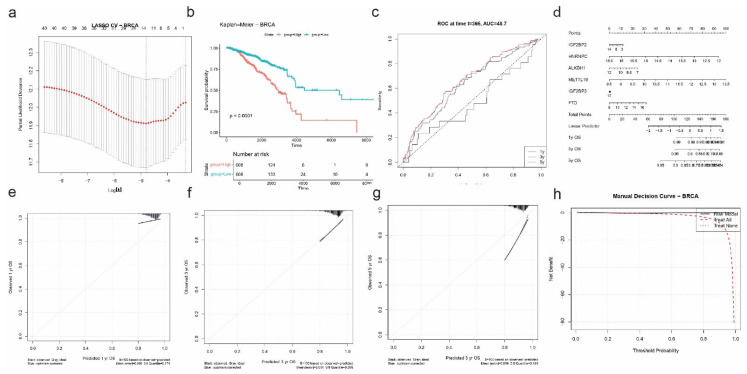
Prognostic model construction and validation for BRCA based on RNA modification-related genes. (**a**) LASSO Cox regression with 10-fold cross-validation identifies optimal λ for feature selection. (**b**) Kaplan–Meier survival curve demonstrates a significant difference in overall survival between high-risk and low-risk groups (*p* < 0.0001). (**c**) Time-dependent ROC analysis at 1, 3, and 5 years; the model shows moderate predictive power (AUC = 48.7 at 1 year). (**d**) Nomogram integrating expression of six selected genes (IGF2BP2, HNRNPC, ALKBH1, METTL16, IGF2BP3, and FTO) to estimate 1-, 3-, and 5-year OS probabilities. (**e**–**g**) Calibration plots for 1-year (**e**), 3-year (**f**), and 5-year (**g**) OS demonstrate close alignment between predicted and observed outcomes, particularly at higher probabilities. (**h**) Decision curve analysis (DCA) assesses the net clinical benefit of the risk model across a range of threshold probabilities.

For breast cancer (BRCA), LASSO Cox regression was performed after univariate screening to identify the most prognostic RNA modification-related genes. Using 10-fold cross-validation (Figure 8a), six genes were selected: IGF2BP2, HNRNPC, ALKBH1, METTL16, IGF2BP3, and FTO. These genes span multiple RNA modification pathways and highlight a complex regulatory network associated with patient survival.

Risk scores were calculated based on a linear combination of the six selected genes, and patients were stratified into high- and low-risk groups. Kaplan–Meier survival analysis showed that the high-risk group had significantly shorter overall survival compared to the low-risk group (*p* < 0.0001; Figure 8b). However, time-dependent ROC analysis revealed modest discriminative ability, with an AUC of 0.487 at 1 year, suggesting that while the gene signature is prognostically informative, its predictive strength is weaker than in other cancers (Figure 8c).

The nomogram constructed for individualized prediction of 1-, 3-, and 5-year OS incorporated the six selected genes (Figure 8d). Calibration plots demonstrated excellent concordance between predicted and observed survival outcomes, particularly at the 3-year timepoint, where the mean absolute error was just 0.004 (Figure 8e). The 1-year and 5-year calibration curves also showed acceptable accuracy, with mean errors of 0.099 and 0.096, respectively.

Finally, decision curve analysis confirmed the potential clinical value of the BRCA-specific risk model. The DCA plot showed a net benefit of the model across a wide range of threshold probabilities compared to the “treat all” or “treat none” approaches (Figure 8f), supporting its relevance in guiding prognostic assessment in breast cancer patients.

#### 2.7.3. Cervical Squamous Cell Carcinoma and Endocervical Adenocarcinoma (CESC)

**Figure 9:**
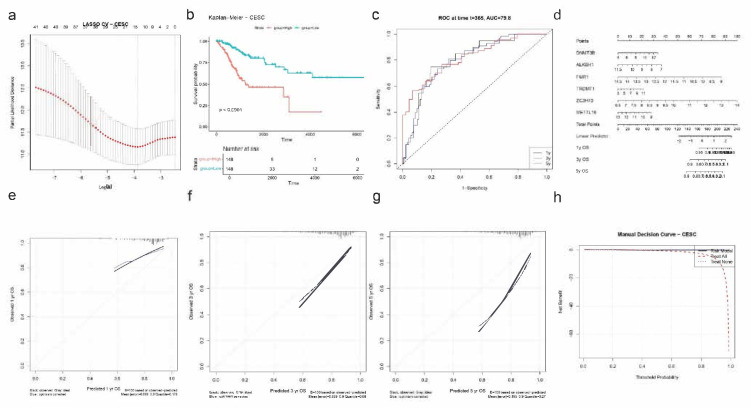
Prognostic model construction and validation for CESC based on RNA modification-related genes. (**a**) LASSO Cox regression with 10-fold cross-validation selects the optimal log(λ) for gene selection. (**b**) Kaplan–Meier survival analysis shows a significant difference in overall survival between high-risk and low-risk patients (*p* < 0.0001). (**c**) Time-dependent ROC curves at 1, 3, and 5 years indicate strong predictive accuracy, with AUC = 79.8 at 1 year. (**d**) Nomogram constructed from six selected genes (DNMT3B, ALKBH1, FMR1, TRDMT1, ZC3H13, and METTL16) to estimate individualized 1-, 3-, and 5-year OS probabilities. (**e**–**g**) Calibration plots for 1-year (**e**), 3-year (**f**), and 5-year (**g**) OS showing high concordance between predicted and actual survival. (**h**) Decision curve analysis (DCA) demonstrates that the model provides net clinical benefit over treat-all or treat-none strategies across a range of threshold probabilities.

To identify prognostic RNA modification regulators in CESC, we applied univariate Cox regression followed by LASSO Cox modeling. Cross-validation identified the optimal lambda that minimized partial likelihood deviance (Figure 9a), yielding six genes: DNMT3B, ALKBH1, FMR1, TRDMT1, ZC3H13, and METTL16.

Patients were stratified into high- and low-risk groups based on the computed risk scores derived from the LASSO model. Kaplan–Meier analysis showed a significant survival difference between risk groups, with the high-risk group exhibiting poorer overall survival (*p* < 0.0001; Figure 9b). ROC curve analysis revealed strong predictive performance at the 1-year mark with an AUC of 0.798, suggesting good discriminative ability of the model (Figure 9c).

A nomogram incorporating the six selected genes was developed to estimate 1-, 3-, and 5-year overall survival probabilities (Figure 9d). Calibration curves indicated excellent agreement between predicted and observed survival at all timepoints, with particularly low error at 3 years (|error| = 0.059) and 1 year (|error| = 0.089) (Figure 9e). The 5-year calibration remained acceptable, with a mean error of 0.195.

Decision curve analysis showed that the model provided net clinical benefit compared to “treat all” and “treat none” strategies across a wide range of threshold probabilities (Figure 9f), supporting its potential clinical application in prognostic assessment for CESC patients.

#### 2.7.4. Colon Adenocarcinoma (COAD)

For colon adenocarcinoma (COAD), univariate Cox regression and LASSO Cox modeling were applied to identify prognostic RNA modification regulators. Although cross-validation was performed and the optimal lambda was determined (Supplementary Figure S1), no genes were selected by the LASSO model at the lambda.min threshold. This result suggests that, within the COAD cohort, the expression levels of the candidate m^6^A/m^5^C/m^1^A regulators included in this analysis may not individually or collectively provide a sufficiently robust prognostic signal.

This finding may reflect biological variability, the relatively small cohort size, or the potential influence of other regulatory mechanisms not captured by this gene set. Further studies incorporating additional molecular layers, such as methylation or protein-level data, may be needed to uncover prognostic markers in COAD.

#### 2.7.5. Glioblastoma Multiforme (GBM)

To construct a prognostic model for GBM, we applied univariate Cox regression followed by LASSO Cox analysis to identify RNA modification-related genes associated with overall survival. The LASSO regression, optimized using 10-fold cross-validation (Supplementary Figure S2a), identified six genes: NSUN2, DNMT3B, HNRNPC, ALKBH1, TRDMT1, and NSUN5.

Patients were stratified into high- and low-risk groups based on the calculated risk score. Kaplan–Meier survival analysis demonstrated significantly reduced survival in the high-risk group (*p* < 0.0001; Supplementary Figure S2b). ROC curve analysis indicated moderate prognostic performance, with an AUC of 0.692 at 1 year, suggesting the model captures relevant biological risk (Supplementary Figure S2c).

A nomogram was constructed to predict 1-, 3-, and 5-year overall survival based on the expression levels of the selected genes (Supplementary Figure S2d). Calibration curves showed acceptable prediction accuracy at 1 year (|error| = 0.081), while prediction error increased substantially at later timepoints (3y: |error| = 0.44; 5y: |error| = 0.507) (Supplementary Figure S2e), reflecting the known poor long-term prognosis in GBM.

Decision curve analysis (Supplementary Figure S2f) confirmed that the model offers a clinical net benefit over the “treat all” and “treat none” strategies, particularly at low to moderate threshold probabilities. This suggests that RNA modification-based signatures may offer clinical utility in GBM despite the aggressive nature of the disease.

#### 2.7.6. Head and Neck Squamous Cell Carcinoma (HNSC)

For HNSC, we conducted LASSO Cox regression following univariate screening to construct a prognostic signature based on RNA modification regulators. Cross-validation (Supplementary Figure S3a) selected six genes: IGF2BP2, HNRNPC, DNMT3A, ZC3H13, NSUN5, and DNMT1, representing multiple RNA modification pathways.

Risk scores were computed and used to stratify patients into high- and low-risk groups. Kaplan–Meier survival analysis demonstrated significantly reduced overall survival in the high-risk group compared to the low-risk group (*p* < 0.0001; Supplementary Figure S3b). Time-dependent ROC analysis showed moderate prognostic accuracy, with an AUC of 0.629 at 1 year (Supplementary Figure S3c).

We constructed a nomogram incorporating the six selected genes to predict 1-, 3-, and 5-year survival (Supplementary Figure S3d). Calibration analysis revealed good agreement between predicted and observed outcomes at 3 and 5 years, with mean absolute errors of 0.09 and 0.184, respectively. The 1-year calibration was less accurate (|error| = 0.205) (Supplementary Figure S3e), possibly due to greater heterogeneity in early clinical outcomes.

Decision curve analysis (Supplementary Figure S3f) indicated that the risk model provides greater net benefit than the “treat all” and “treat none” approaches across a wide range of threshold probabilities, supporting its potential utility in prognostic assessment for HNSC patients.

#### 2.7.7. Kidney Renal Clear Cell Carcinoma (KIRC)

In KIRC, LASSO Cox regression was performed following univariate analysis to construct a prognostic model based on RNA modification regulators. Using 10-fold cross-validation (Supplementary Figure S4a), six genes were selected: YTHDC2, IGF2BP2, TRMT6, ALKBH1, HNRNPA2B1, and METTL16, spanning m6A, m1A, and m5C modification pathways.

Patients were stratified into high- and low-risk groups according to their calculated risk scores. Kaplan–Meier analysis demonstrated a highly significant survival difference, with high-risk patients showing markedly worse overall survival than those in the low-risk group (*p* < 0.0001; Supplementary Figure S4b). ROC analysis indicated strong predictive ability, with a 1-year AUC of 0.773 (Supplementary Figure S4c).

A nomogram was developed to estimate 1-, 3-, and 5-year overall survival based on the six selected genes (Supplementary Figure S4d). Calibration plots showed excellent accuracy, particularly at 1 year (mean |error| = 0.039), while prediction remained acceptable at 3 years (|error| = 0.11) and 5 years (|error| = 0.246) (Supplementary Figure S4e).

Decision curve analysis showed a clear clinical net benefit of the model over “treat all” or “treat none” strategies across a range of threshold probabilities (Supplementary Figure S4f), supporting the potential use of RNA modification-based signatures in clinical prognostication of KIRC patients.

#### 2.7.8. Kidney Renal Papillary Cell Carcinoma (KIRP)

For KIRP, we applied univariate Cox regression followed by LASSO Cox modeling to develop a prognostic signature. Using 10-fold cross-validation, the optimal lambda was selected (Supplementary Figure S5a), yielding six RNA modification-related genes: YTHDC2, IGF2BP2, DNMT3B, TRMT6, HNRNPC, and NSUN5.

Patients were classified into high- and low-risk groups based on their risk scores derived from the LASSO model. Kaplan–Meier analysis revealed a significant survival difference, with poorer survival in the high-risk group (*p* = 0.00035; Supplementary Figure S5b). ROC analysis demonstrated strong model performance, with a 1-year AUC of 0.861, suggesting high discriminatory power (Supplementary Figure S5c).

We constructed a nomogram integrating the selected genes to predict 1-, 3-, and 5-year overall survival (Supplementary Figure S5d). Calibration plots showed very good predictive accuracy at 3 years (|error| = 0.044) and acceptable error at 1 year (|error| = 0.107) and 5 years (|error| = 0.16) (Supplementary Figure S5e).

Decision curve analysis confirmed that the risk model provided a clinical net benefit across a wide range of threshold probabilities compared to standard strategies (Supplementary Figure S5f). These results suggest that epitranscriptomic regulators may serve as valuable prognostic markers in KIRP and support their potential role in individualized patient stratification.

### 2.8. Single-Cell Expression Analysis of Prognostic RNA Modification Genes via TISCH2

To dissect the cell-type-specific expression of RNA modification-related prognostic markers identified through LASSO Cox regression, we conducted single-cell RNA-seq (scRNA-seq) analysis across six tumor types using the TISCH2 platform. This approach enabled precise mapping of gene expression across major cellular compartments in the tumor microenvironment, including immune, stromal, and malignant cell types. Supplementary Figures S6–S11 illustrate the UMAP cell type clusters, gene expression overlays, and violin plots for each tumor.

#### 2.8.1. Single-Cell Characterization of RNA Modification Genes in BLCA

To further investigate the cellular origin and distribution of prognostic RNA modification regulators in bladder cancer, we analyzed single-cell RNA sequencing data from the BLCA_GSE130001 dataset using the TISCH2 platform. Major cell populations identified by UMAP projection included epithelial cells (tumor-derived), fibroblasts, myofibroblasts, and endothelial cells (Supplementary Figure S6a). Epithelial cells constituted the majority of the tumor microenvironment.

We examined the single-cell expression profiles of the six RNA modification regulators selected from the LASSO Cox model (NSUN2, IGF2BP2, YTHDC1, ALKBH5, ZC3H13, and TRDMT1) across these cellular compartments. UMAP-based expression overlays revealed that NSUN2, YTHDC1, ALKBH5, ZC3H13, and TRDMT1 were predominantly expressed within epithelial clusters, consistent with a tumor-intrinsic regulatory role (Supplementary Figure S6b). Notably, IGF2BP2 expression was more restricted, showing focal enrichment primarily in stromal regions, particularly fibroblasts.

Violin plot analysis confirmed these patterns and quantitatively demonstrated that NSUN2, ALKBH5, YTHDC1, and ZC3H13 were highly expressed in epithelial cells, while IGF2BP2 was enriched in fibroblasts and TRDMT1 showed modest expression in myofibroblasts (Supplementary Figure S6c).

These findings support the tumor-intrinsic role of several RNA modification regulators in bladder cancer while also implicating IGF2BP2 and TRDMT1 in stromal remodeling and tumor–stroma interactions. The cell-type-specific expression patterns highlight the potential relevance of RNA epitranscriptomic machinery in both cancer progression and microenvironmental dynamics.

#### 2.8.2. Single-Cell Characterization of RNA Modification Genes in BRCA

To elucidate the cellular distribution of prognostic RNA modification regulators in breast cancer, we analyzed the BRCA_EMTAB8107 single-cell RNA-seq dataset using the TISCH2 platform. UMAP clustering revealed a heterogeneous tumor microenvironment composed of malignant epithelial cells, fibroblasts, myofibroblasts, endothelial cells, CD8+ T cells (including exhausted CD8Tex), B cells, plasma cells, mast cells, monocytes/macrophages, and proliferating T cells (Supplementary Figure S7a).

Among the six LASSO-selected genes (HNRNPC, ALKBH1, METTL16, IGF2BP2, IGF2BP3, and FTO), HNRNPC exhibited the most abundant and widespread expression across nearly all annotated cell types, with notable enrichment in malignant, stromal, and immune populations (Supplementary Figure S7b). In contrast, IGF2BP2, IGF2BP3, and METTL16 were sparsely expressed, while ALKBH1 and FTO showed weak-to-moderate expression primarily in fibroblasts and myeloid cells.

Violin plots confirmed that HNRNPC was consistently and highly expressed, particularly in malignant cells, CD8+ T cells, and proliferating T cells, whereas the remaining genes displayed low or restricted expression profiles (Supplementary Figure S7c).

These results suggest that HNRNPC may play a broadly functional role in breast cancer biology, while other regulators demonstrate more context-specific or limited cellular activity.

#### 2.8.3. Single-Cell Characterization of RNA Modification Genes in CESC

To investigate the cell-type-specific expression of RNA modification-related prognostic markers in cervical cancer, we analyzed the CESC_GSE168652 dataset using the TISCH2 single-cell platform. UMAP clustering identified distinct cellular populations, including malignant epithelial cells, fibroblasts, smooth muscle cells (SMCs), endothelial cells, CD8+ T cells, monocytes/macrophages, and endometrial stromal cells (Supplementary Figure S8a).

Expression patterns of six LASSO-selected RNA modification genes (ALKBH1, DNMT3B, FMR1, METTL16, TRDMT1, and ZC3H13) were evaluated. Among these, FMR1 and ZC3H13 showed moderate expression across multiple cell types, with enrichment observed particularly in fibroblasts, malignant cells, and immune subsets (Supplementary Figure S8b). In contrast, DNMT3B, ALKBH1, METTL16, and TRDMT1 displayed uniformly low expression across all clusters.

Violin plots confirmed that FMR1 exhibited the most notable expression in malignant, mono/macrophage, and CD8+ T-cell compartments, whereas other genes showed either minimal or highly restricted expression (Supplementary Figure S8c).

These results suggest that within the CESC tumor microenvironment, FMR1 and ZC3H13 may serve as the most biologically relevant RNA modification regulators, potentially contributing to tumor–stroma or tumor–immune interactions.

#### 2.8.4. Single-Cell Resolution of RNA Modification Regulator Expression in KIRC

To examine cell-type-specific expression of RNA modification regulators in kidney renal clear cell carcinoma (KIRC), we analyzed the KIRC_GSE159115 dataset via the TISCH2 platform. UMAP clustering revealed diverse cellular populations, including malignant epithelial cells, endothelial cells, pericytes, monocytes/macrophages, plasma cells, erythroblasts, and CD8+ T cells (Supplementary Figure S9a).

Of the six prognostic RNA modification genes identified via LASSO Cox modeling (HNRNPA2B1, ALKBH1, METTL16, IGF2BP2, TRMT6, and YTHDC2), only HNRNPA2B1 exhibited consistently high expression across nearly all annotated cell types. Notably, it was strongly enriched in endothelial cells, epithelial clusters, malignant cells, and immune populations, suggesting a widespread regulatory role in KIRC (Supplementary Figure S9b).

In contrast, IGF2BP2, TRMT6, METTL16, ALKBH1, and YTHDC2 showed sparse and low-level expression, primarily restricted to epithelial and malignant clusters. Violin plots quantitatively confirmed these findings, with HNRNPA2B1 showing the highest and most uniform expression, while the other regulators remained weakly expressed (Supplementary Figure S9c).

These results indicate that HNRNPA2B1 likely plays a dominant and widespread functional role within the KIRC tumor microenvironment, influencing both malignant and stromal compartments. The limited expression of the other regulators suggests more restricted, possibly context-dependent, functions.

#### 2.8.5. Single-Cell RNA-Seq Analysis of Prognostic RNA Modification Regulators in HNSC

To investigate the cell-type-specific expression of RNA modification regulators in head and neck squamous cell carcinoma (HNSC), we analyzed the GSE139324 dataset via the TISCH2 platform. UMAP clustering revealed a diverse cellular composition comprising CD4+ conventional T cells (CD4Tconv), CD8+ T cells (CD8T and CD8Tex), natural killer (NK) cells, B cells, regulatory T cells (Tregs), proliferating T cells (Tprolif), plasma cells, dendritic cells (DCs), mast cells, and monocytes/macrophages (Mono/Macro) (Supplementary Figure S10a).

Expression overlays of six RNA modification genes selected by the LASSO Cox model (DNMT1, DNMT3A, HNRNPC, IGF2BP2, NSUN5, and ZC3H13) demonstrated variable distribution across the tumor microenvironment (Supplementary Figure S10b). HNRNPC showed high and broad expression across almost all immune subsets, with notable enrichment in CD8Tex, Tregs, Tprolif, and malignant-like cell populations.

DNMT1 and DNMT3A displayed more limited expression patterns, with DNMT1 more strongly expressed in plasma and Tprolif clusters, and DNMT3A weakly expressed across a range of immune cell types. The violin plots supported these observations quantitatively (Supplementary Figure S10c). HNRNPC was highly expressed across all major immune and stromal cell populations, peaking in CD4Tconv, CD8Tex, and monocyte/macrophage clusters. In contrast, IGF2BP2, NSUN5, and ZC3H13 showed consistently low expression across all lineages. DNMT1 exhibited moderate but variable expression in plasma, Tregs, and Tprolif cells.

These findings highlight HNRNPC as a ubiquitously and highly expressed RNA modification regulator in HNSC, potentially contributing broadly to tumor–immune interactions. Meanwhile, other genes in the prognostic signature appear to have more restricted or context-dependent expression profiles in the HNSC tumor microenvironment.

#### 2.8.6. Single-Cell Analysis of RNA Modification Regulator Expression in Glioma

To explore the cell-type-specific expression of prognostic RNA modification regulators in glioma, we analyzed the Glioma_GSE141383 dataset. UMAP clustering revealed five major cell populations: malignant cells, oligodendrocytes, fibroblasts, endothelial cells, and monocytes/macrophages (Supplementary Figure S11a).

Expression mapping of the LASSO-selected genes (HNRNPC, NSUN2, NSUN5, DNMT3B, TRDMT1, and ALKBH1) revealed that HNRNPC was robustly expressed across all cell types, with notable enrichment in malignant, fibroblast, and oligodendrocyte clusters (Supplementary Figure S11b).

Violin plots further confirmed high and broadly distributed HNRNPC expression. NSUN5 showed moderate expression with slight enrichment in fibroblasts and oligodendrocytes, while NSUN2 displayed weak, diffuse expression across malignant and immune compartments. TRDMT1 exhibited restricted expression limited to a small subset of oligodendrocytes. DNMT3B was minimally expressed, with sparse signal across all cell types (Supplementary Figure S11c).

Overall, these results highlight HNRNPC as a widely active epitranscriptomic regulator in the glioma microenvironment, suggesting a potential pan-lineage regulatory role, while other markers appear more context- or cell-type specific.

## 3. Discussion

RNA modifications are dynamic and reversible chemical alterations that play a crucial role in various cellular and molecular processes, including RNA stability and translation [86]. These modifications are regulated by three major classes of proteins: “writers,” which catalyze the addition of chemical marks; “erasers,” which remove them; and “readers,” which recognize and propagate downstream effects of modified RNAs [87]. The influence of post-transcriptional modifications on cancer immunity has been suggested by different studies [76,88]. However, beyond classical transcriptional and translational control, the regulatory landscape of immune checkpoints and other immune molecules extends into the field of epitranscriptomics.

Our findings support a model in which RNA modifications, particularly m^6^A, act as fine-tuners of CD70, CD80, and TIGIT expression across multiple cancer types. This aligns with emerging evidence suggesting that m^6^A modifications enhance immune evasion in some tumors while promoting antigen presentation in others [30,89,90]. Notably, our study revealed intriguing insights into epigenetic regulators such as METTL3 and METTL14, which function as “writers” by installing m^6^A marks on RNA. We found that METTL3 and METTL14 were upregulated in LUAD, BRCA, and BLCA. A literature review revealed both supportive and contradictory findings: Guo et al. confirmed elevated METTL3 levels in LUAD and BLCA and linked METTL3 expression to immune cell abundance in LUAD, underscoring its role in the tumor microenvironment [91]. Additional studies further corroborated the upregulation of METTL3, describing its underlying mechanisms and consequences [92,93,94,95]. Conversely, our findings regarding METTL14 expression differed from previous reports as four studies indicated reduced METTL14 levels in BRCA and BLCA, contradicting our results [96,97,98,99].

Through PPI analysis, we identified WTAP as a key interactor of METTL3 and METTL14, forming the METTL3–METTL14–WTAP complex, which plays a crucial role in m6A deposition. Our comprehensive analysis revealed WTAP overexpression in LUAD and STAD, a finding corroborated by Lei et al. [100]. The co-occurrence of these three “writers” in the tumor microenvironment suggests their potential as prognostic and therapeutic biomarkers, a topic we explore further.

Our analysis also highlighted the impact of m^6^A “erasers,” particularly FTO and ALKBH5. We observed FTO overexpression in LUAD and PRAD, aligning with its established role in immune evasion via downregulation of immune-stimulating genes. This suggests that FTO overexpression in lung and prostate cancers may impair CD70/CD80-mediated immune activation [68,69]. ALKBH5, another demethylase, exhibited downregulation in ESCA and HNSC in our analysis. However, Wei et al. [101] reported contrasting findings, suggesting ALKBH5 overexpression in these cancers. Our results imply that ALKBH5 downregulation may impair RNA stability and reduce co-stimulatory molecule expression, such as CD70, CD80, and TIGIT, thereby contributing to immune suppression.

In addition to the expression levels of RNA modification enzymes, the deposition and function of epitranscriptomic marks such as m^6^A are influenced by intrinsic RNA sequence motifs and the chromatin landscape. Li et al. (2024) [102] demonstrated that specific consensus regions within RNA sequences, characterized by distinct motifs (e.g., RRACH), are critical for accurate identification of m6A sites. Their model, M6A-DCR, uses graph contrastive clustering to enhance interpretability and identify sequence-driven deposition hotspots [102]. Furthermore, chromatin accessibility plays a pivotal role in regulating gene expression by modulating transcriptional activity. As reviewed by Klemm et al. (2019), accessible chromatin states facilitate both transcription and epigenetic regulator recruitment, including RNA methylation complexes [103]. Therefore, both RNA motifs and chromatin structure must be considered when interpreting the impact of RNA modifications on immune checkpoint gene regulation as these elements may modulate m^6^A deposition and transcript fate in a context-dependent manner.

The emerging role of RNA modifications in regulating immune checkpoint genes presents promising avenues for cancer immunotherapy. Among these, m^6^A, the most prevalent internal modification in eukaryotic mRNA, plays a central role in post-transcriptional gene regulation. Increasing evidence supports its function as a modulator of key immune regulatory molecules, including CD70, CD80, and TIGIT, influencing cancer immune evasion. Given that CD70 and CD80 are essential for T-cell activation, while TIGIT acts as an inhibitory receptor mediating immune suppression, the epitranscriptomic control of their expression holds high therapeutic relevance.

Based on our findings, we propose that targeting RNA methylation patterns opens new therapeutic possibilities. Inhibitors of FTO, such as CS1 and CS2, have demonstrated the ability to sensitize tumor cells to T-cell-mediated cytotoxicity by downregulating immune checkpoint molecules like LILRB4, a member of the leukocyte immunoglobulin-like receptor subfamily B, which has been implicated in immune evasion in AML [104,105,106]. Su et al. showed that CS1 and CS2 suppressed LILRB4 expression in AML cells, reducing tumor immune evasion [68]. We hypothesize that in solid tumors where immune checkpoint inhibitors such as PD-L1 and TIGIT are overexpressed, CS1 and CS2 could similarly reduce their levels, enhancing monoclonal antibody therapies targeting PD-L1 and other ICI therapies.

With continuous technological advancements, novel inhibitors continue to emerge. In 2020, Li et al. identified ALK-04, an ALKBH5-specific inhibitor, which reduced Treg and MDSC infiltration, thereby enhancing the efficacy of anti-PD-1 treatment [107]. Additionally, STM2457, a METTL3/METTL14 inhibitor, exhibited potent antileukemic effects in preclinical AML models, highlighting the feasibility of targeting m^6^A writers for cancer therapy [108]. However, a significant challenge remains: these inhibitors have demonstrated efficacy primarily in hematologic malignancies, while the precise role of m6A modifications in solid tumors remains unclear. The complexity and heterogeneity of the tumor microenvironment further complicate their therapeutic targeting [30]. Future research should focus on elucidating the context-specific effects of RNA modifications in solid tumors to harness their full therapeutic potential. To complement our mechanistic findings, we developed a prognostic model based on the expression of four RNA modification regulators: YTHDF3, RBM15B, IGF2BP2, and TRMT61A. These genes represent distinct epitranscriptomic mechanisms, spanning m^6^A, m^5^C, and m^1^A pathways. IGF2BP2, an m^6^A “reader,” is upregulated in head and neck squamous cell carcinoma (HNSC), where it correlates with poor prognosis and regulates pathways related to cell proliferation and immune modulation [109]. In gastric and pancreatic cancers, IGF2BP2 stabilizes oncogenic mRNAs and promotes glutamine metabolism, suggesting similar roles in the aggressive behavior seen in cancers like COAD and KIRP [110,111]. YTHDF3 has been implicated in immune evasion and metastatic progression. In non-small cell lung cancer, YTHDF3 promotes immune suppression via PD-L1 upregulation [112], while in gastric cancer, it activates PI3K/AKT signaling and alters the tumor immune microenvironment [113]. Its known role in breast cancer brain metastases may reflect similar metastatic potential in BRCA cases within our dataset. RBM15B, a component of the m^6^A writer complex, shows cancer-type specific functions. In triple-negative breast cancer, it promotes proliferation through metabolic reprogramming [114]. Conversely, in other cancers like uveal melanoma, it may play a protective role through immune checkpoint regulation. TRMT61A, an m^1^A writer, regulates lipid metabolism and immune signaling. Additionally, TRMT61A modulates cholesterol biosynthesis and supports CD8+ T-cell responses [115], suggesting an intersection with tumor immunology in these cancers. Together, these genes reflect diverse biological roles tied to oncogenesis and immune modulation in cancers closely matching those in our cohort. Their inclusion in a survival model with high AUC values and clinical utility supports the idea that RNA modification regulators are not only mechanistically relevant but also clinically actionable.

To further dissect the biological relevance of RNA modification regulators in individual tumor contexts, we performed LASSO-based survival modeling across eight TCGA cancer types, identifying unique prognostic signatures tailored to each tumor’s microenvironment. In bladder cancer (BLCA), NSUN2, IGF2BP2, YTHDC1, ALKBH5, TRDMT1, and ZC3H13 were identified, with NSUN2—a 5-methylcytosine (m5C) writer—previously shown to promote tumorigenesis and cancer stemness by stabilizing CCND1 mRNA and driving epithelial–mesenchymal transition. TRDMT1 and ZC3H13 have also been implicated in modulating mRNA splicing and stability, suppressing tumor progression in urological cancers [116]. In breast cancer (BRCA), the model selected HNRNPC, IGF2BP2, IGF2BP3, FTO, METTL16, and ALKBH1. Among them, HNRNPC has been experimentally validated as a regulator of dsRNA-induced interferon signaling and immune activation, suggesting it may promote immune suppression and cancer progression via alternative splicing and RNA processing [117]. In cervical cancer (CESC), FMR1 and ZC3H13 emerged as key regulators. FMR1 has recently been implicated in post-transcriptional control of immune checkpoint genes and RNA transport, providing a novel link between RNA modification and tumor immunity [118]. In glioblastoma (GBM), NSUN2, DNMT3B, NSUN5, TRDMT1, ALKBH1, and HNRNPC were selected. NSUN5, in particular, is known to regulate ribosomal stress responses and translational control, facilitating glioma adaptation to stress and correlating with poor prognosis [119]. In head and neck squamous cell carcinoma (HNSC), the presence of HNRNPC, DNMT1, DNMT3A, ZC3H13, NSUN5, and IGF2BP2 highlights potential cross-talk between DNA and RNA methylation in immune modulation. IGF2BP2, a well-characterized m6A reader and oncogenic mRNA stabilizer, has repeatedly been associated with proliferation and poor survival outcomes in various cancers including HNSC [120]. In kidney renal clear cell carcinoma (KIRC), HNRNPA2B1, another m6A reader, showed strong prognostic power and is known to modulate immune checkpoint transcript isoforms through splicing regulation [121]. Finally, in kidney papillary carcinoma (KIRP), the signature included YTHDC2, IGF2BP2, DNMT3B, TRMT6, HNRNPC, and NSUN5. YTHDC2 regulates mRNA decay and export, while TRMT6, a tRNA methyltransferase, may influence antigen presentation via translational efficiency, marking it as a novel biomarker in immune-enriched tumors [122,123]. Collectively, these tumor-specific prognostic signatures validate and expand upon known functions of RNA modification regulators, aligning with mechanistic insights from the literature and offering opportunities for biomarker-guided precision oncology.

To complement bulk RNA-based prognostic modeling, we performed single-cell RNA-seq analysis across six TCGA cancer types to investigate the cellular context and heterogeneity of RNA modification gene expression. Using TISCH2, we mapped the LASSO-identified prognostic markers across tumor, immune, and stromal compartments, providing crucial insights into their functional localization and potential cellular mechanisms.

In multiple tumor types, notably BRCA, HNSC, and glioma, HNRNPC emerged as the most broadly and abundantly expressed regulator across malignant and immune cells, consistent with its known role in m6A-mediated alternative splicing and RNA maturation. In BRCA, its expression extended to CD8+ and proliferating T cells, suggesting immunomodulatory involvement. Similarly, in HNSC and glioma, HNRNPC was strongly expressed in monocytes/macrophages and T-cell subsets, indicating a pan-lineage regulatory function possibly supporting immune suppression or evasion mechanisms. These findings reinforce HNRNPC’s central role in tumor–immune cross-talk and highlight it as a promising pan-tumor biomarker for both biological function and clinical prediction [124].

Conversely, regulators such as IGF2BP2, METTL16, and FTO demonstrated highly context-specific and sparse expression across tumor types. In BRCA and KIRC, IGF2BP2 was weakly detected in malignant and stromal cells, contrasting with its prognostic significance at the bulk level [125]. This discrepancy may reflect post-transcriptional regulation or low cell-type-specific expression not captured in averaged bulk data. Similarly, ZC3H13 and TRDMT1, despite inclusion in multiple prognostic signatures (e.g., BLCA and CESC), showed moderate-to-low expression in fibroblasts and immune cells, supporting roles in stromal remodeling rather than direct tumor cell regulation [126].

In KIRC and CESC, single-cell analysis revealed novel spatial patterns. HNRNPA2B1 was consistently and strongly expressed across endothelial, malignant, and immune cells in KIRC, aligning with its predicted role in splicing and checkpoint gene regulation. In contrast, FMR1 and ZC3H13 showed moderate expression in fibroblasts and immune compartments in CESC, potentially linking RNA stabilization to stromal–immune interactions.

Interestingly, glioma-specific regulators such as NSUN5 and TRDMT1 exhibited localized enrichment in oligodendrocytes and fibroblasts, suggesting their roles in neuro-glial remodeling and stress adaptation [119]. Meanwhile, DNMT3B, a DNA methyltransferase with emerging RNA-related functions, remained sparsely expressed in all tumors, raising questions about its prognostic role and calling for further validation.

These findings underscore the importance of integrating single-cell data to validate and contextualize bulk RNA-based biomarkers. The observed variability in expression across cell types highlights that prognostic relevance may stem not only from gene expression magnitude but also from cell-type specificity. Furthermore, widespread expression of regulators like HNRNPC and HNRNPA2B1 supports their functional significance across diverse tumor microenvironments, while sparse or restricted expression of others suggests niche or indirect regulatory functions.

Overall, our single-cell analysis enhances the biological interpretability of RNA modification-related prognostic models, revealing which regulators act in malignant, stromal, or immune niches. This cell-type resolution is essential for guiding precision medicine strategies targeting the epitranscriptome, and supports the future development of cell-type-informed RNA methylation inhibitors tailored to specific tumor ecosystems.

We are aware that this study is based on RNA-seq data and does not include direct measurement of m^6^A, m^1^A, or m^5^C modifications on CD70, CD80, or TIGIT. The presence or absence of these modifications cannot be confirmed without epitranscriptomic profiling methods such as MeRIP-seq, m^1^A-quant-seq, or bisulfite sequencing. Therefore, our findings are correlative and should be considered hypothesis-generating, pending future validation through biochemical and molecular assays.

Finally, we would also like to point out that we acknowledge that RNA-seq-based analysis cannot directly measure methylation activity, protein expression, or biochemical function. Therefore, all proposed mechanistic links remain hypotheses to be tested by future studies, employing functional assays, such as MeRIP-seq, or reporter-based mRNA stability models. Moreover, this study is exploratory in nature and based on bulk RNA expression. We acknowledge that our conclusions are intended as hypotheses to guide future work integrating transcriptomic, epitranscriptomic, and proteomic layers to clarify the casual role of RNA modifications in immune checkpoint regulation.

## 4. Materials and Methods

### 4.1. Data Acquisition

Gene expression and clinical data were obtained from The Cancer Genome Atlas (TCGA) via the UCSC Xena platform (https://xenabrowser.net; accessed on 10 April 2025), as well as through the Gene Set Cancer Analysis (GSCA) platform (http://bioinfo.life.hust.edu.cn/GSCA; accessed on 10 April 2025), SangerBox 2 (http://sangerbox.com/; accessed on 10 April 2025), and the Genotype Tissue Expressiom (GTex) Portal. These platforms provide harmonized RNA-seq datasets, immune infiltration estimates, and mutation annotations across diverse cancer types [127,128,129,130,131].

From TCGA, we included only primary tumor samples with complete clinical metadata, including survival status, overall survival (OS) time, age, sex, and TNM stage. Samples missing any of these elements were excluded to ensure clinical consistency. Gene expression matrices were log_2_-transformed using the formula log_2_(x + 0.001) to stabilize variance and reduce right-skew in highly expressed genes.

Using GSCA, we extracted mRNA expression profiles and immune cell infiltration data based on RNA-seq deconvolution. In addition, we explored RNA modification patterns across cancer types using curated gene sets for m^6^A, m^5^C, and m^1^A regulators. 

SangerBox 2 was employed for further data normalization, visualization, and reannotation where needed, taking advantage of its graphical interface and automated scripts for pan-cancer analysis. To focus the study on epitranscriptomic regulation, we compiled a comprehensive list of m^6^A, m^5^C, and m^1^A RNA modification regulators, including writers, readers, and erasers, based on published studies [29,30]. Expression correlations between these regulators and CD70, CD80, and TIGIT were computed using Pearson’s correlation coefficient, across tumor types.

### 4.2. m^6^A/m^1^A/m^5^C Gene Expression

We investigated the differential expression of the readers, writers, and erasers across 14 cancer types, including bladder cancer, BLCA (n = 19); breast cancer, BRCA (n = 114); colon adenocarcinoma, COAD (n = 26); esophageal carcinoma, ESCA (n = 11); head and neck squamous cell carcinoma, HNSC (n = 43); kidney chromophobe, KICH (n = 25); kidney renal clear cell carcinoma, KIRC (n = 72); kidney renal papillary cell carcinoma, KIRP (n = 32); liver hepatocelullar carcinoma, LIHC (n = 50); lung adenocarcinoma, LUAD (n = 58); lung squamous cell carcinoma, LUSC (n = 51); prostate adenocarcinoma, PRAD (n = 52); stomach adenocarcinoma, STAD (n = 32); and thyroid adenocarcinoma, THCA (n = 59).

By comparing the expression patterns of genes encoding readers (YTHDF1-3, YTHDC1-3, ALYREF, IGF2BP1, LRPPRC, FMR1, HNRNPA2B1, HNRNPC, and ELAVL1), writers (TRMT61A, TRMT10C, TRMT61B, NSUN5, DNMT1, DNMT3B, NOP2, NSUN2, NSUN4, TRDNMT1, DNMT3A, NSUN7, NSUN3, NSUN6, METTL3, METTL14, CLL1, RBM15B, ZC3H13, WTAP, RBM15, and KIAA1429), and erasers (ALKBH1, ALKBH3, ALKBH5, TET2, and FTO), between normal and tumor tissues, we inferred the potential dysregulation of these RNA modifications and their potent role in tumorigenesis. The *p*-value was estimated by *t*-test and further adjusted by FDR. FDR values ≤ 0.05 were considered statistically significant [132].

### 4.3. Gene Ontology (GO) and Kyoto Encyclopedia of Genes and Genomes (KEGG) Enrichment Analyses

GO enrichment was used to explore the biological processeses (BPs), cellular component (CC), and molecular functions (MFs) that our associated m^6^A/m^1^A/m^5^C regulated genes take part in. KEGG enrichment analysis was used to explore the molecular pathways that these genes take part in and their resepective role [133,134,135].

### 4.4. The Protein Network of m^6^A/m^1^A/m^5^C Regulated Genes

To investigate functional associations among RNA modification-related proteins, we constructed a protein–protein interaction (PPI) network, using the STRING database (https://string-db.org/; accessed on 10 April 2025). The gene list for RNA modification-related enzymes was used as input. A confidence score threshold of ≥0.4 was set to filter reliable interactions, ensuring a balanced inclusion of known and predicted associations. The network was visualized and analyzed within the STRING’s web-based interface, using Cytoscape 3.10.3 for enhanced graphical represenatation. Functional enrichment analysis was conducted for GO terms and KEGG pathways, identifying significantly enriched BP, MF, and pathways related to RNA modifications and cancer. STRING assigns a confidence score to each interaction, which depicts the likelihood of a true functional association. Enrichment analyses were evaluated with the Fischer’s exact test and FDR correction (*q* ≤ 0.05) to adjust for multiple testing [136,137].

### 4.5. TCGA-Based Expression Profiling and Survival Modeling

RNA-Seq expression data (STAR—Counts workflow) were retrieved from TCGA for eight cancer types: BLCA, BRCA, cervical squamous cell carcinoma and endocervical adenocarcinoma (CESC), COAD, glioblastoma multiforme (GBM), HNSC, KIRC, and KIRP. Corresponding normal tissue samples were included where available. Raw count matrices were transformed using log_2_(x + 0.001) and filtered to include genes encoding m^6^A, m^5^C, and m^1^A RNA modification regulators (writers, readers, and erasers).

Sample type (tumor or normal) was annotated based on the TCGA barcode structure, using the 14–15th character to identify sample type (“01” for tumor and “11” for solid normal tissue).

Prognostic modeling was conducted using gene expression data from tumor samples only. We implemented a dual-layer approach, applying both pan-cancer and cancer-specific (per-cancer) modeling strategies. This design allowed us to detect generalizable signatures across multiple tumor types as well as tumor-type-specific patterns with clinical implications.

First, we performed univariate Cox proportional hazards regression across the full pan-cancer cohort using the survival R package (v. 4.1.1) to identify RNA modification-related genes significantly associated with overall survival (OS). Genes with *p* < 0.05 were retained for further analysis. We then applied LASSO Cox regression using the glmnet package (v.4.1-9) to build a multigene prognostic model. LASSO was selected for its ability to manage high-dimensional gene expression data, select informative features, and prevent overfitting. Ten-fold cross-validation was used to select the optimal penalty parameter (lambda.min) that minimized the cross-validated partial likelihood deviance.

In parallel, we repeated the same modeling pipeline separately for each of the eight selected cancer types: BLCA, BRCA, CESC, COAD, GBM, HNSC, KIRC, and KIRP. For each cancer, we independently conducted univariate Cox regression followed by LASSO Cox modeling with 10-fold cross-validation. This cancer-stratified approach enabled the identification of tumor-type-specific prognostic gene signatures and improved model stability and interpretability by accounting for tumor heterogeneity.

For both pan-cancer and per-cancer models, a risk score was calculated per patient using the expression of selected genes weighted by their LASSO-derived coefficients. Patients were dichotomized into high- and low-risk groups based on the median risk score.

Prognostic models were evaluated using Kaplan–Meier survival curves, log-rank test, and time-dependent ROC analysis at 1-, 3-, and 5-year time points using the timeROC package (v. 0.4). For each model, a nomogram was constructed with the rms package (v. 8.0-0), and calibration was assessed using 100 bootstrap resamples.

Clinical applicability was further evaluated through decision curve analysis (DCA) using the ggDCA (v. 1.1) and rmda (v. 1.6) packages. Due to limited long-term follow-up, 5-year predictions were assessed by ROC but excluded from calibration and DCA. While external validation was not performed, the combined pan-cancer and per-cancer analysis improves the robustness, specificity, and translational relevance of the prognostic models.

### 4.6. Single-Cell RNA-Seq Analysis

To complement the bulk RNA-seq analyses, we explored the tumor microenvironment–specific expression of RNA modification-related genes and immune checkpoints using the Tumor Immune Single-cell Hub (TISCH2) web platform (http://tisch.comp-genomics.org/; accessed on 5 May 2025). TISCH2 provides preprocessed single-cell RNA-seq datasets with annotated cell types across various human cancers.

For each of the eight cancer types analyzed (BLCA, BRCA, CESC, COAD, GBM, HNSC, KIRC, and KIRP), we identified representative scRNA-seq datasets and examined the expression patterns of key m^6^A, m^5^C, and m^1^A regulators, across annotated cell populations such as malignant cells, T cells, B cells, macrophages, and endothelial cells.

Gene expression visualizations were generated directly through the TISCH2 browser. These analyses enabled us to assess whether selected prognostic genes were preferentially expressed in specific cellular compartments within the tumor microenvironment, thus providing additional biological context for the bulk survival models.

## 5. Conclusions

In conclusion, we highlight the critical role of the m^6^A, m^5^C, and m^1^A RNA modifications in regulating CD70, CD80, and TIGIT across multiple solid tumors. By combining epitranscriptomics data with functional enrichment and survival modeling, we show that RNA modification enzymes not only modulate immune-related gene expression but also serve as potential biomarkers for patient prognosis. By constructing a robust four-gene prognostic signature involving YTHDF3, RBM15B, IGF2BP2, and TRMT61A, we demonstrate that RNA modification profiles can accurately stratify patients into risk groups with distinct overall survival outcomes. The performance of this model across eight cancer types underscores the translational promise of epitranscriptomic markers in both mechanistic understanding and personalized oncology. Altogether, our study bridges the gap between the mechanistic regulation of immune checkpoints and their clinical utility, offering novel insights into how the epitranscriptome can be leveraged to improve cancer prognosis and potentially enhance immunotherapeutic strategies. 

## Data Availability

Genomic data were extracted from TCGA (https://portal.gdc.cancer.gov/, accessed on 1 February 2025) and GTEx (https://www.gtexportal.org/home/, accessed on 1 February 2025).

## Supplementary Materials

The following supporting information can be downloaded at https://www.mdpi.com/article/10.3390/ijms26125772/s1.
