# Supplementary material for: Epitranscriptomics Regulation of CD70, CD80, and TIGIT in Cancer Immunity
Source: Int J Mol Sci. 2025 Jun 16;26(12):5772. doi: 10.3390/ijms26125772 (PMC12193086; doi:10.3390/ijms26125772)
Supplement: Supplementary file 1 [file ijms-26-05772-s001.zip › ijms-3627266-supplementary.pdf]

Supplementary Figures

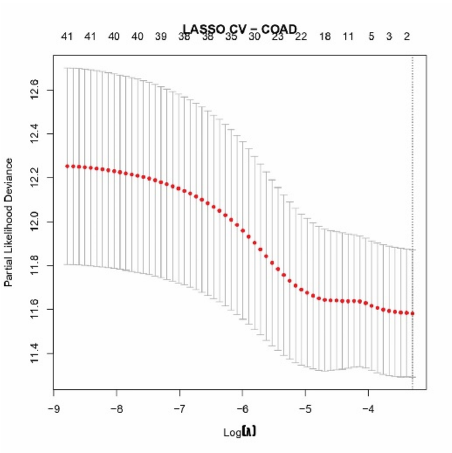

Figure S1. **LASSO regression analysis for COAD.** LASSO Cox regression with 10-fold cross-validation was conducted to identify prognostic RNA modification-related genes. The plot shows the partial likelihood deviance versus log(λ). Despite model training, no genes met the selection threshold under optimal λ, and therefore no risk model or downstream validation (KM, ROC, nomogram) was constructed for COAD.

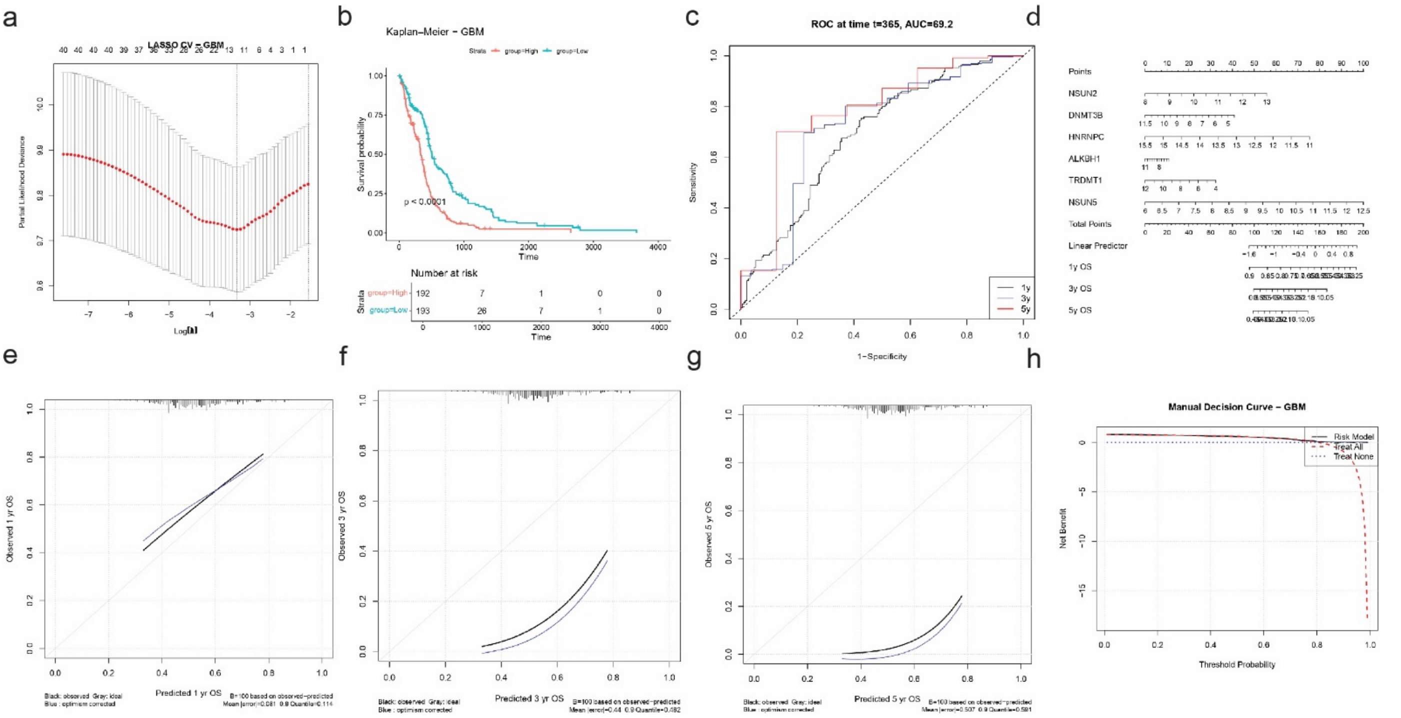

Figure S2. **Prognostic model construction and validation for GBM based on RNA modification-related genes.** (a) LASSO Cox regression with 10-fold cross-validation identifies the optimal value of log(λ) used to select the most prognostic genes. (b) Kaplan–Meier survival curve shows significantly reduced overall survival in the high-risk group compared to the low-risk group ($p < 0.0001$). (c) Time-dependent ROC curves at 1, 3, and 5 years demonstrate good predictive performance of the risk model (AUC = 69.2 at 1 year). (d) Nomogram incorporating six genes (NSUN2, DNMT3B, HNRNPC, ALKBH1, TRDMT1, NSUN5) to estimate 1-, 3-, and

5-year OS probabilities. (e–g) Calibration plots for 1-year (e), 3-year (f), and 5-year (g) OS showing acceptable agreement between predicted and observed outcomes. (h) Decision curve analysis (DCA) reveals net clinical benefit of the risk model across a range of threshold probabilities when compared to treat-all and treat-none strategies.

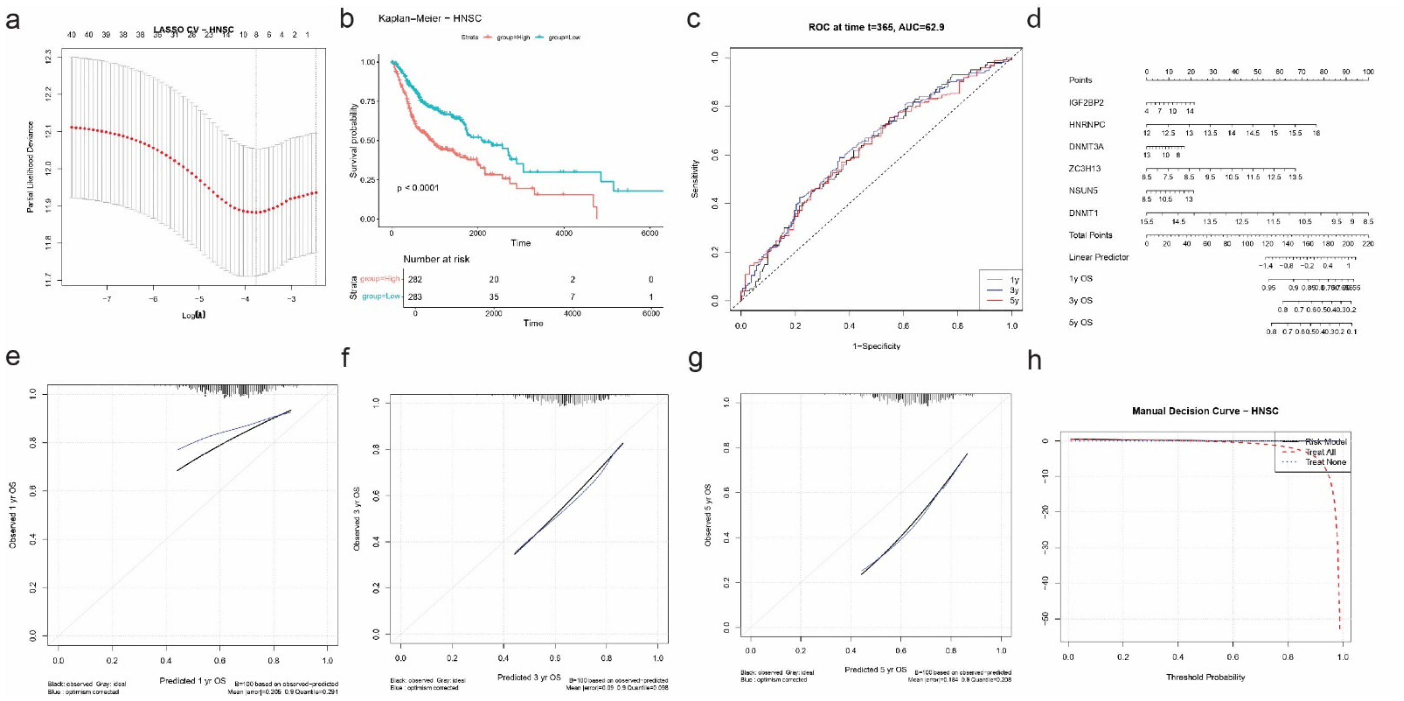

Figure S3. **Prognostic model construction and validation for HNSC based on RNA modification-related genes.** (a) LASSO Cox regression with 10-fold cross-validation identifies the optimal penalty (log[λ]) for selecting survival-associated genes. (b) Kaplan–Meier survival curve reveals a significant difference in overall survival between high-risk and low-risk patients ($p < 0.0001$). (c) Time-dependent ROC analysis for 1-, 3-, and 5-year overall survival shows moderate predictive capability (AUC = 62.9 at 1 year). (d) Nomogram including six selected genes (IGF2BP2, HNRNPC, DNMT3A, ZC3H13, NSUN5, TRDMT1) enables individualized estimation of 1-, 3-, and 5-year survival probabilities. (e–g) Calibration plots for 1-year (e), 3-year (f), and 5-year (g) OS demonstrate good agreement between predicted and actual survival, particularly in the mid-to-high probability range. (h) Decision curve analysis (DCA) evaluates clinical usefulness, showing that the model provides net benefit across a wide range of threshold probabilities.

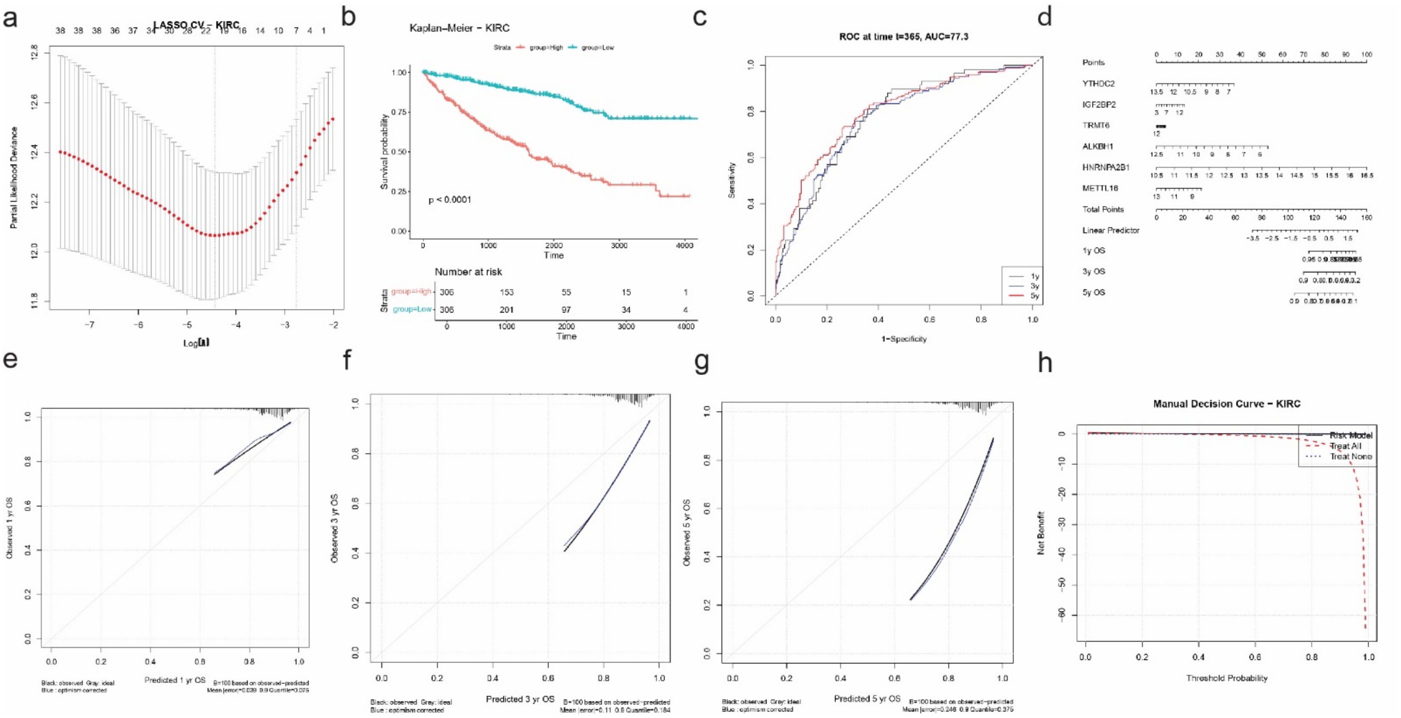

Figure S4. **Prognostic model construction and validation for KIRC based on RNA modification-related genes.** (a) LASSO Cox regression with 10-fold cross-validation selects the optimal log(λ) for model sparsity and performance. (b) Kaplan–Meier survival analysis indicates significantly lower survival in the high-risk group compared to the low-risk group ($p < 0.0001$). (c) Time-dependent ROC curves for 1-, 3-, and 5-year overall survival show strong predictive performance (AUC = 77.3 at 1 year). (d) Nomogram incorporating six selected genes (YTHDC2, IGF2BP2, TRMT6, ALKBH1, HNRNPA2B1, METTL16) provides individualized survival predictions at 1, 3, and 5 years. (e–g) Calibration plots for 1-year (e), 3-year (f), and 5-year (g) OS demonstrate excellent concordance between predicted and observed outcomes. (h) Decision curve analysis (DCA) shows that the risk model yields a favorable net clinical benefit across a wide range of threshold probabilities, outperforming treat-all and treat-none strategies.

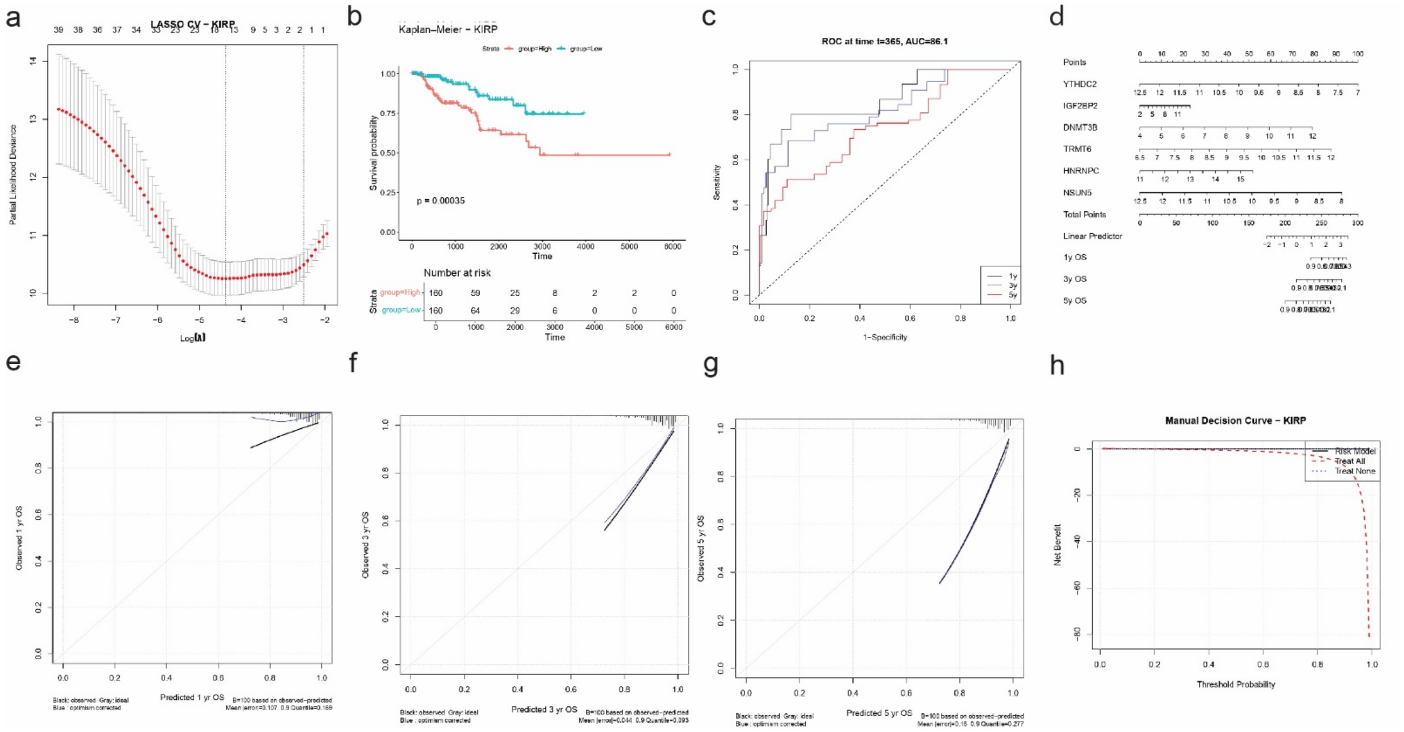

Figure S5. **Prognostic model construction and validation for KIRP based on RNA modification-related genes.** (a) LASSO Cox regression with 10-fold cross-validation identifies the optimal penalty parameter log(λ) for selecting survival-associated genes. (b) Kaplan–Meier survival curve reveals a significant overall survival difference between the high-risk and low-risk groups (p = 0.00035). (c) Time-dependent ROC curves for 1-, 3-, and 5-year OS demonstrate excellent model performance (AUC = 86.1 at 1 year). (d) Nomogram based on six selected genes (YTHDC2, IGF2BP2, DNMT3B, TRMT6, HNRNPC, NSUN5) enables prediction of individualized 1-, 3-, and 5-year survival probabilities. (e–g) Calibration plots for 1-year (e), 3-year (f), and 5-year (g) OS confirm the strong agreement between predicted and observed survival outcomes. (h) Decision curve analysis (DCA) supports the clinical utility of the model, offering a higher net benefit across thresholds compared to treat-all or treat-none strategies.

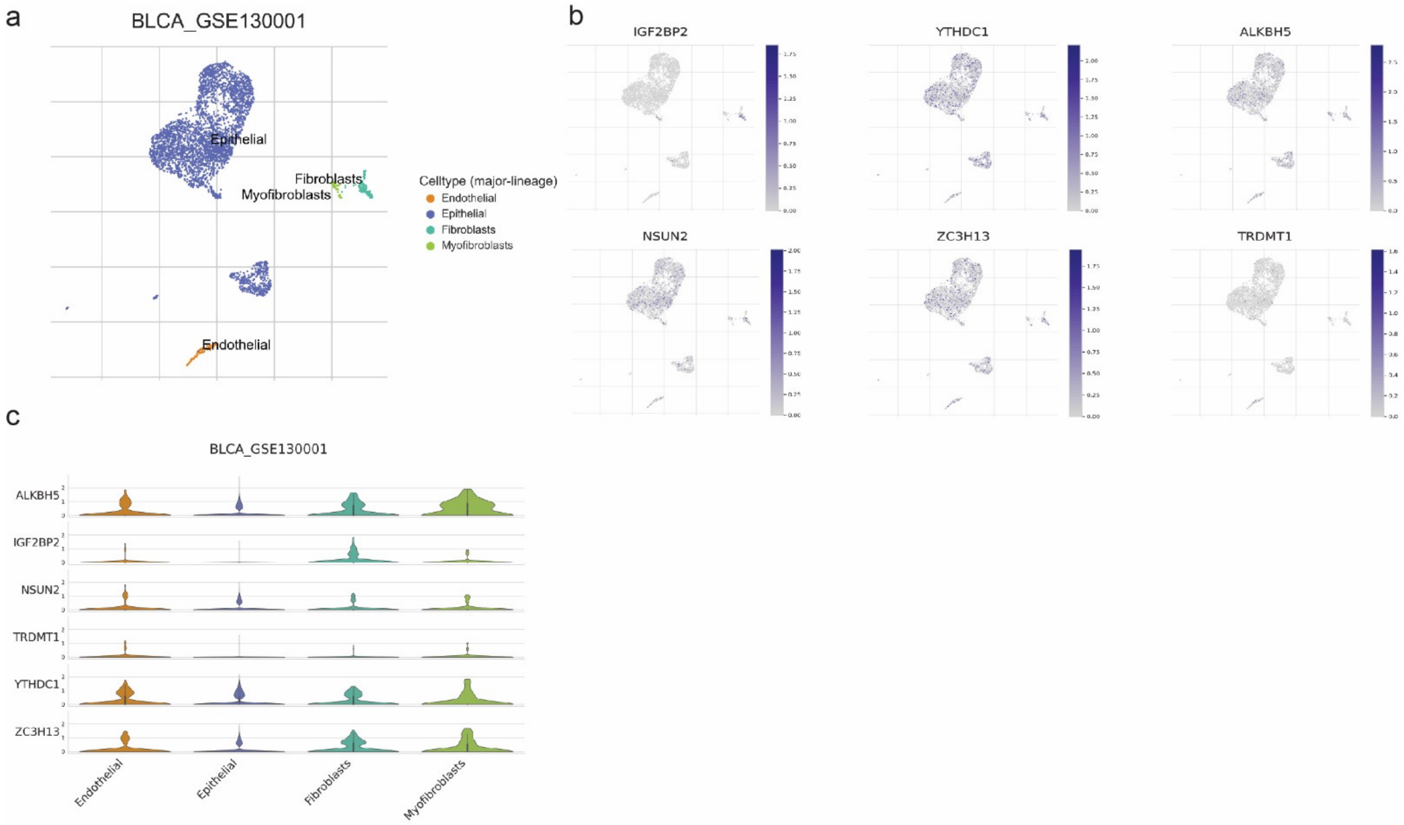

Figure S6. **Single-cell expression analysis of RNA modification genes in bladder cancer.** (a) UMAP plot showing major cell lineages: Epithelial (blue), Endothelial (orange), Fibroblasts (teal), and Myofibroblasts (green). (b) UMAP feature plots illustrating the expression of six RNA modification-related genes (IGF2BP2, YTHDC1, ALKBH5, NSUN2, ZC3H13, TRDMT1) across the identified cell populations. (c) Violin plots showing the distribution of expression levels for these genes across the four major cell types, highlighting lineage-specific expression differences.

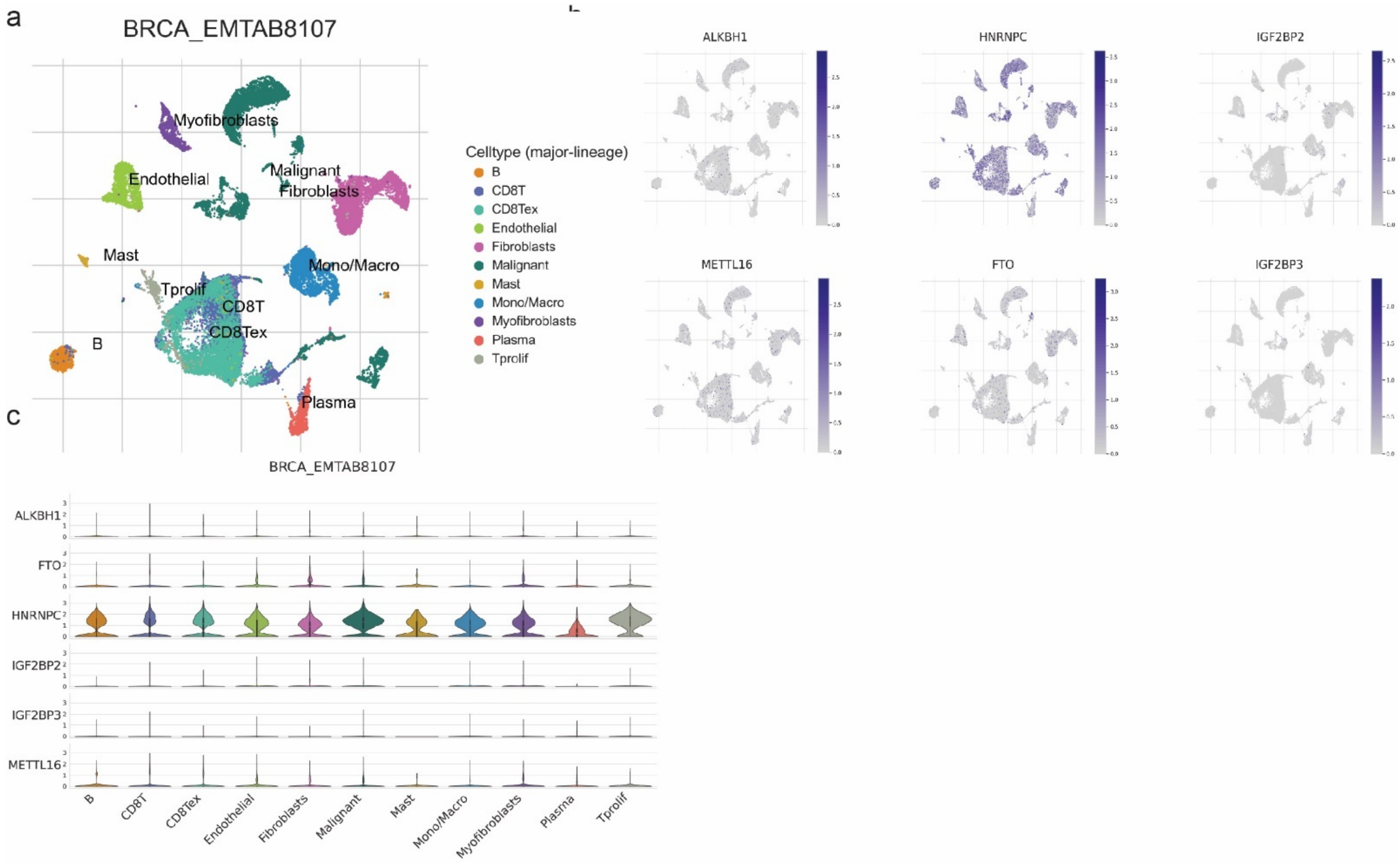

Figure S7. Single-cell expression landscape of RNA modification genes in breast cancer. (a) UMAP plot showing major cell types in the breast cancer tumor microenvironment, including CD8T, CD8Tex, Endothelial, Fibroblasts, Malignant, Mast, Mono/Macrophages, Myofibroblasts, Plasma, B cells, and proliferating T cells (Tprolif). (b) UMAP feature plots illustrating expression of six RNA modification-related genes (ALKBH1, HNRNPC, IGF2BP2, IGF2BP3, METTL16, FTO) across the identified cell types. Color intensity reflects normalized expression levels. (c) Violin plots depicting the distribution of expression levels for the same genes across the annotated cell populations, highlighting differences in cell type–specific expression patterns.

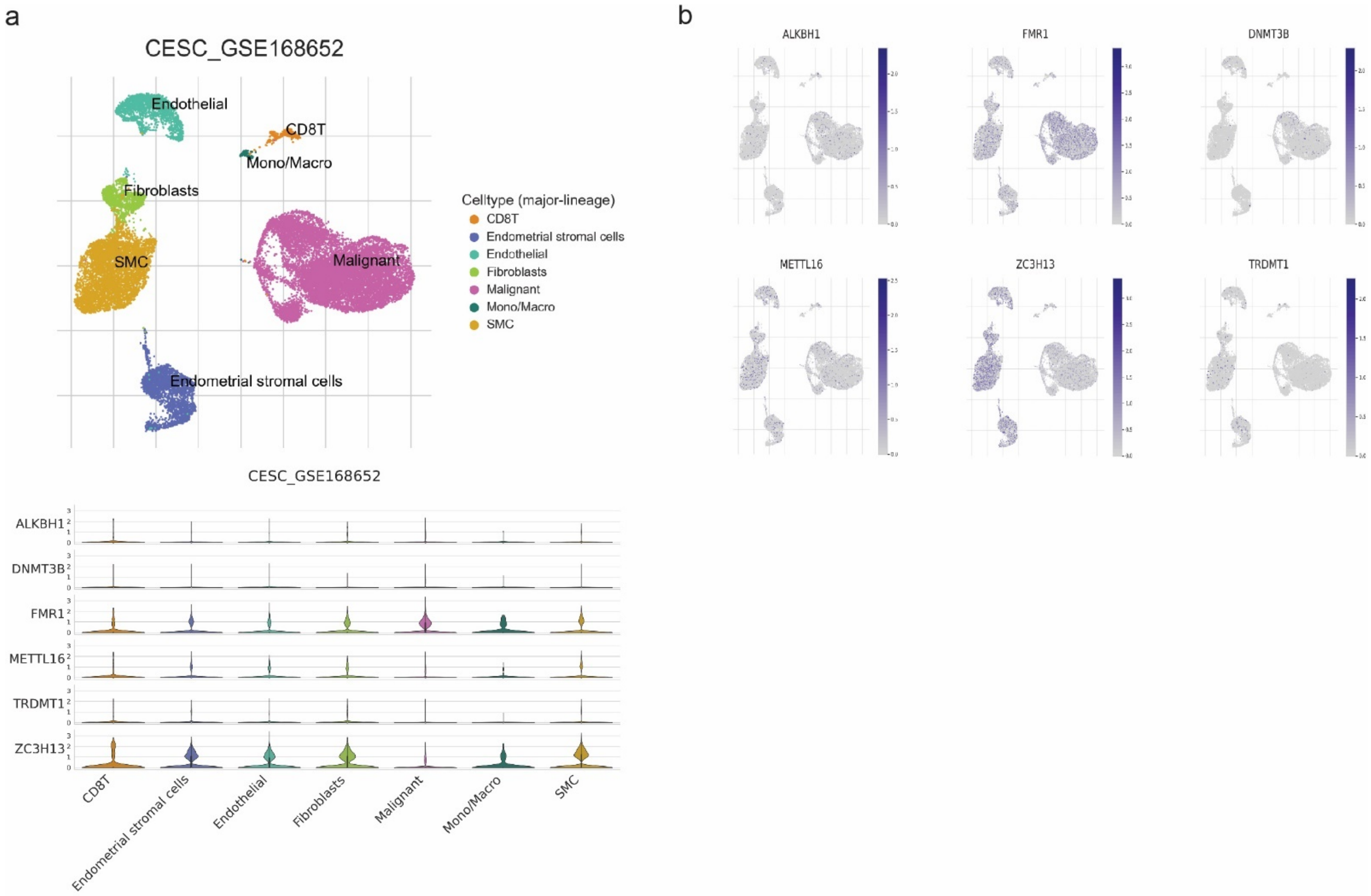

Figure S8. Single-cell expression analysis of RNA modification genes in cervical cancer. (a) UMAP plot depicting major cell lineages, including CD8 T cells, Endometrial stromal cells, Endothelial cells, Fibroblasts, Malignant cells, Monocytes/Macrophages, and Smooth Muscle Cells (SMC). (b) UMAP feature plots showing the expression of six RNA modification-related genes (ALKBH1, FMR1, DNMT3B, METTL16, ZC3H13, TRDMT1) across the identified cell types. Expression levels are represented by a gradient from gray (low) to blue (high). (c) Violin plots displaying the distribution of gene expression levels for these six genes across all annotated cell populations, revealing cell type–specific expression patterns

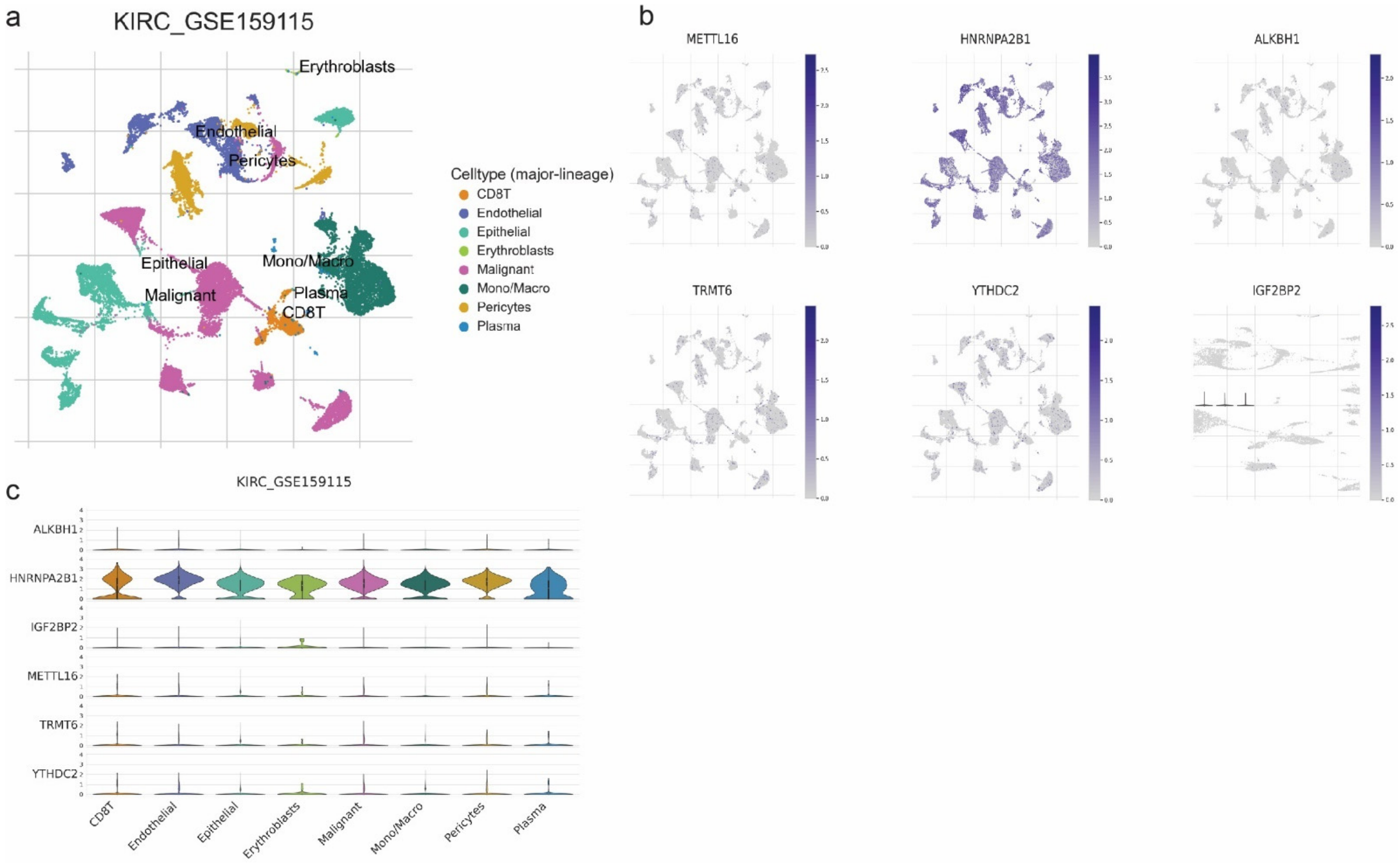

Figure S9. **Single-cell expression profiling of RNA modification genes in kidney cancer.** (a) UMAP plot showing major cell populations, including CD8 T cells, Endothelial cells, Epithelial cells, Erythroblasts, Malignant cells, Monocytes/Macrophages, Pericytes, and Plasma cells. (b) UMAP feature plots illustrating the expression of six RNA modification genes (METTL16, HNRNPA2B1, ALKBH1, TRMT6, YTHDC2, IGF2BP2) across cell types. Expression intensity is visualized from gray (low) to blue (high). (c) Violin plots depicting gene expression distributions across annotated cell types, highlighting differential expression of RNA modification genes within the KIRC tumor microenvironment.

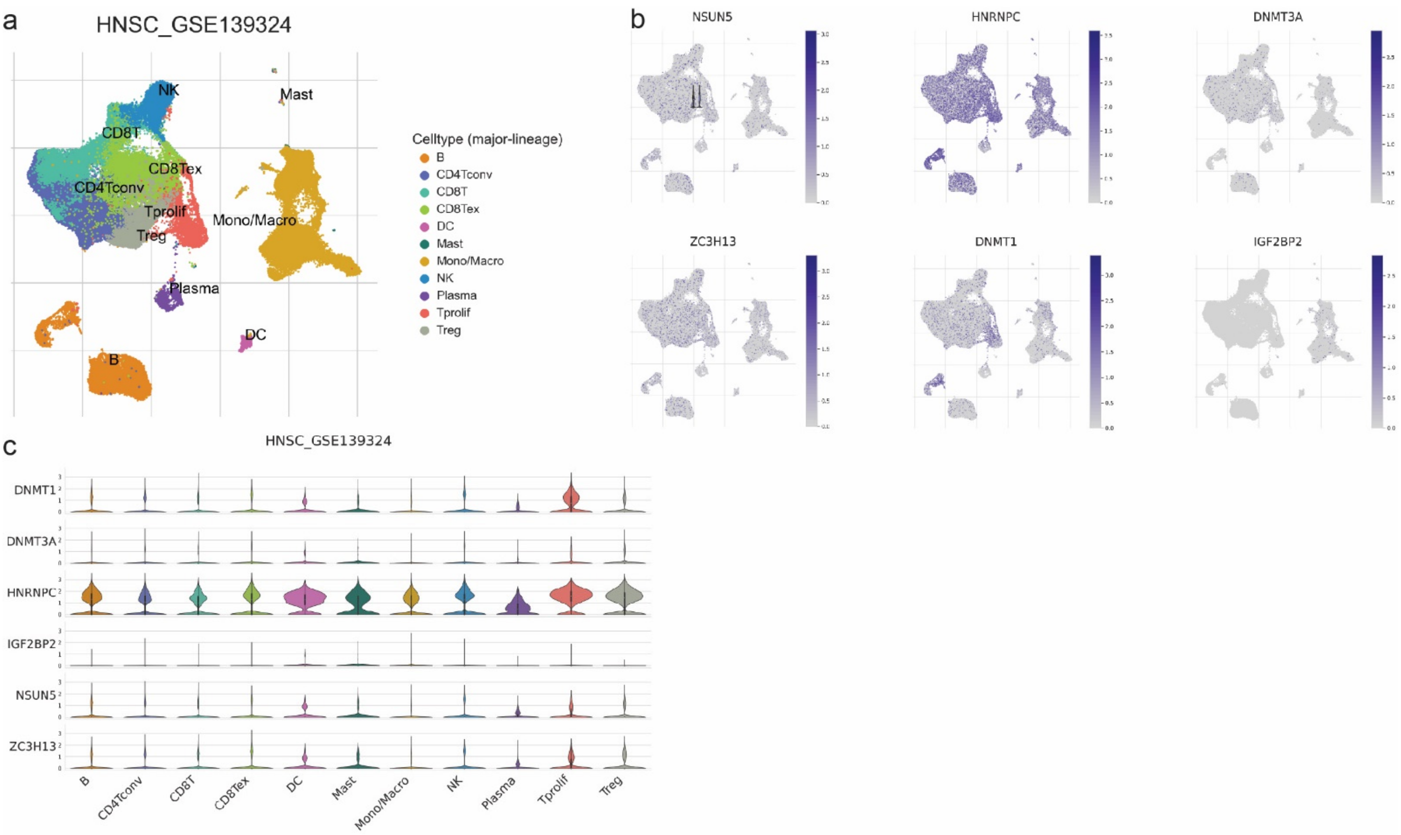

Figure S10. **Single-cell expression landscape of RNA modification genes in HNSC.** (a) UMAP plot showing major cell populations including B cells, CD4$^+$ T conventional (CD4Tconv), CD8$^+$ T cells (CD8T), exhausted CD8$^+$ T cells (CD8Tex), dendritic cells (DC), mast cells, monocytes/macrophages (Mono/Macro), natural killer (NK) cells, plasma cells, proliferating T cells (Tprolif), and regulatory T cells (Treg). (b) UMAP feature plots displaying the expression of six RNA modification genes (NSUN5, HNRNPC, DNMT3A, ZC3H13, DNMT1, IGF2BP2) across the identified cell types. Expression levels are represented by a gray-to-blue color gradient. (c) Violin plots illustrating the distribution of expression for these genes across annotated cell types, highlighting lineage-specific transcriptional activity.

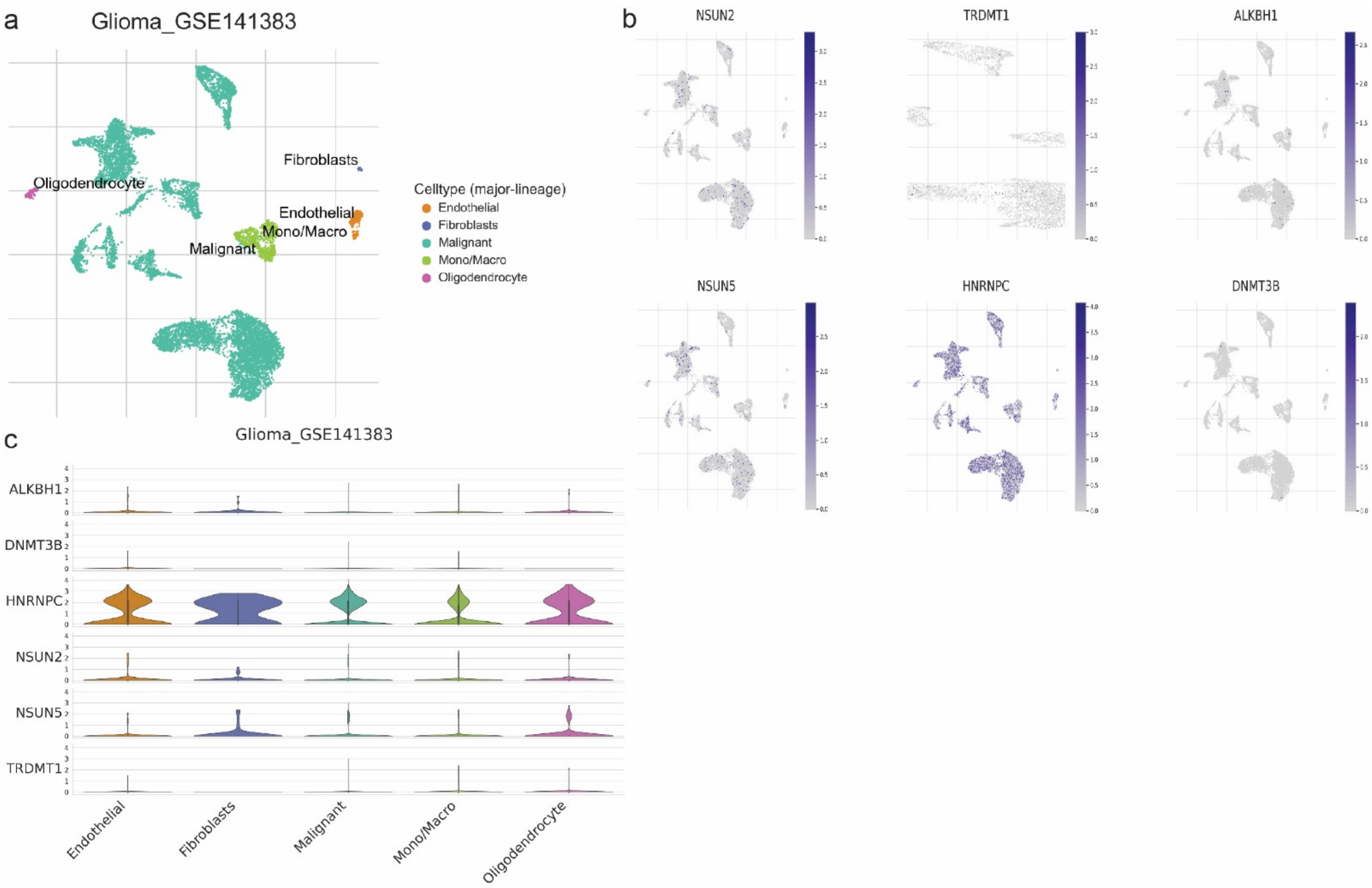

Figure S11. Single-cell expression landscape of RNA modification genes in glioma. (a) UMAP plot showing major cell populations including Endothelial cells, Fibroblasts, Malignant cells, Monocytes/Macrophages, and Oligodendrocytes. (b) UMAP feature plots displaying the expression of six RNA modification genes (NSUN2, TRDMT1, ALKBH1, NSUN5, HNRNPC, DNMT3B) across the identified cell types. Expression levels are represented by a gray-to-blue color gradient. (c) Violin plots illustrating the distribution of expression for these genes across annotated cell types, highlighting lineage-specific transcriptional activity.
